# Hidden costs and unmet supportive care needs among individuals with experience of breast cancer and their carers in the United Kingdom

**DOI:** 10.1038/s44276-025-00172-z

**Published:** 2025-08-19

**Authors:** Szeyi Ng, Lucy S. Kilburn, Hilary Stobart, Lesley Stephen, Indrani S. Bhattacharya, David A. Cameron, Rebecca Lewis, Stuart A. McIntosh, Carlo Palmieri, Catherine Towns, Charlotte E. Coles, Judith M. Bliss

**Affiliations:** 1 Clinical Trials and Statistics Unit at The Institute of Cancer Research, London, UK; 2 Independent Cancer Patients’ Voice, London, UK; 3 Make 2nds Count, Edinburgh, UK; 4 Cambridge University Hospitals NHS Foundation Trust, Cambridge, UK; 5 University of Edinburgh Cancer Centre, Institute of Genetics and Cancer, Edinburgh, UK; 6 Patrick G Johnston Centre for Cancer Research, Queen’s University Belfast, Belfast, UK; 7 Institute of Systems, Molecular and Integrative Biology, Molecular and Clinical Cancer Medicine, University of Liverpool, Liverpool, UK; 8 The Clatterbridge Cancer Centre NHS Foundation Trust, Liverpool, UK; 9 University of Cambridge, Cambridge, UK; 10 Department of Oncology, University of Cambridge, Cambridge, UK

## Abstract

**Background:**

The impact of cancer transcends physical health, affecting mental wellbeing, financial stability, and ability to perform daily tasks, influencing not only patients but also the broader community.

**Methods:**

Online anonymous surveys (24/01/2023–03/03/2023) were disseminated via charities to individuals treated for breast cancer in the UK and their carers. Multivariable ordered logistic regression models were used to investigate demographic, cancer-related and employment factors associated with physical, wellbeing and financial Quality-of-Life (QoL).

**Results:**

470 and 136 participants reported primary (PBC) and metastatic (MBC) breast cancer, respectively. 27% PBC and 35% MBC participants reported experience of financial problems. 17% PBC and 47% MBC participants reported trouble fulfilling caring responsibilities at the time of survey completion. For PBC participants, reports of financial problems were associated with difficulties seeking help for physical or wellbeing issues, which were associated with worse physical and wellbeing QoL. Financial problems, and other challenges were more commonly reported among MBC participants. These factors may impact QoL similarly, so there was no evidence of specific explanatory factors for MBC participants.

**Conclusions:**

Better understanding of wider impact of breast cancer could lead to better policy and support. Future clinical trials should incorporate more comprehensive assessment of breast cancer’s wider effects.

## Background

The impact of cancer transcends physical health, affecting mental wellbeing, financial stability, social functioning, and ability to carry out daily tasks over an extended period [1–4]. Patients often experience psychological distress, including loss of confidence in performing routine tasks and anxiety about the uncertainty of mortality [1, 3, 5]. Long-term physical and emotional issues may also diminish patients’ capacity to fulfil work, caring or domestic responsibilities [6]. While public healthcare or insurance may cover direct medical costs (e.g. treatment, laboratory testing), direct non-medical costs (e.g. transportation, special equipment) and indirect costs (e.g. loss of income) are often not covered for patients and their care givers [7]. Together, these costs contribute to financial toxicity, the negative impact of cancer-related expenses on the financial wellbeing of patients and their families. This may lead to social and financial hardships that directly or indirectly impact their families and the broader community [8–11].

The existing literature is limited and predominantly focuses on the direct medical costs associated with breast cancer care, where patients experiencing financial toxicity may delay, reduce, or discontinue treatment, resulting in poorer survival outcomes [12, 13]. As part of the Lancet Breast Cancer Commission [14], we examined financial toxicity in the United Kingdom, a high-income country with a National Health Service that provides healthcare free at the point of use. We conducted the Costs and Supportive Care in Breast Cancer (CASCARA) pilot study using online anonymous population-based surveys scoping financial toxicity and supportive care needs, which include physical, emotional, psychosocial, and practical aspects of care during and after treatment, for UK individuals with experience of breast cancer and their carers. Our research aims to provide insights into individual experience of the wider influence of breast cancer in the UK and calls for the development of new metrics to measure holistic quality of life after breast cancer. Our objective is to characterise factors impacting financial toxicity and scope physical and well-being supportive care needs amongst UK individuals with breast cancer and their carers. High level descriptive results were published in the Lancet Breast Cancer Commission report [14]. This manuscript provides more detailed descriptive statistics and explores relationships between various factors, including baseline information such as age, challenges such as financial problems and endpoints such as Quality of Life (QoL).

## Methods

### Study design

The study methodology was briefly described in the Lancet Breast Cancer Commission report and more details about the development of CASCARA patient survey and carer survey are given here [14]. The surveys were designed by researchers from the Lancet Breast Cancer Commission and The Institute of Cancer Research Clinical Trials & Statistics Unit (ICR-CTSU), in collaboration with volunteers with lived experience of primary and metastatic breast cancer (PBC and MBC), through discussion groups [14]. NHS ethics approval is not required for this study and Higher Education Institution approval was provided by The Royal Marsden Committee for Clinical Research (ICR-CTSU/2022/10084).

Firstly, a set of discussion groups were conducted including volunteers from locations across the UK (e.g. King’s Lynn, Swansea, Belfast) with lived experience of PBC and MBC, to identify relevant themes. Recruitment posters for these discussion groups were disseminated across various charities located throughout the UK (see Acknowledgements). In total, 16 volunteers of various ages and backgrounds were selected as participants and were offered compensation for their time at £25/hour. Each volunteer was invited to attend two separate two-hour long virtual discussion groups led by a patient advocate partner (LS or HS) and a researcher (CEC) from the Lancet Breast Cancer Commission. The first set of discussion groups were used to discuss participants’ experience of hidden costs and supportive care needs of breast cancer. As recommended by our patient advocates, the first set of discussion groups were held separately for volunteers with experience in PBC and MBC.

Secondly, using insights from these discussion groups and reference to previous publications, two surveys were designed: one for people who had a diagnosis of breast cancer (patient survey) (Supplementary Material 1), and another for people who have experience of caring for someone with breast cancer (carer survey) (Supplementary Material 2). Demographic and employment details, caring and supportive care needs were included in both patient and carer surveys. Financial situation, access to clinical trials and attitudes to terminologies were only included in the patient survey.

Thirdly, the surveys were sent to the volunteers ahead of the second set of discussion groups and reviewed and refined in the second set of discussion groups. The same volunteers from the first set of discussion groups participated in the second round, but this time, participants with experience of PBC and MBC were not separated. Finally, volunteers from ICR-CTSU conducted testing to ensure the questions were clear, user-friendly and that the online surveys operated smoothly.

### Procedure

The surveys were held online on the Jisc platform, open from 24/01/2023 and closed at midnight 03/03/2023. Completion and submission of the survey was considered consent for participation in the study [14].

People who have had a diagnosis of breast cancer and received their treatment in the UK were eligible to the patient survey. Relatives or friends who care or have cared for someone with breast cancer who received their treatment in the UK were eligible to the carer survey [14].

The surveys were disseminated via Breast Cancer Now (a UK breast cancer charity), other patient-focussed platforms and external groups and charities (see Acknowledgements). Advertisements for CASCARA were shared on charity websites, shared via email newsletters or posters to emailing lists, or shared via social media [14].

### Statistical methods

Participants with experience of primary breast cancer (PBC participants) and metastatic breast cancer (MBC participants) answered the same questions, but the two groups were analysed separately as they formed two distinct cohorts given the likelihood of different lived experiences. Participants with more than one diagnosis of breast cancer or recurrence, were asked to provide information related to their most recent diagnosis of disease. Some questions about patient experience referred to diagnosis and survey completion. Duration between the two timepoints had no set minimum or maximum and was included as a factor in the secondary analysis.

Detailed descriptive data were reported using frequencies and percentages for categorical variables and using median and interquartile range for continuous variables in the patient and carer surveys. The secondary analysis investigated the relationships between different factors within the patient survey. Univariable ordered logistic regression was first applied. Factors with statistically significance results (*p* < 0.05) in univariable regression and low collinearity with other factors (Cramer’s V statistic < 0.4) were then used to build multivariable ordered logistic regression models. Factors with high collinearity were selected with statistical and clinical input. Benjamini-Hochberg adjusted *p*-value with α = 0.05 was provided to adjust for multiple testing. Primary endpoints were physical, wellbeing and financial quality of life (QoL) and were rated on a 4-point scale (Very poorly/Poorly/Well/Very Well). Primary and exploratory endpoints, covariates and their corresponding questions are listed in Supplementary Table A1. For categorical variables, categories with responses fewer than 5% of total responses were merged with another appropriate category with similar interpretation. ‘Prefer not to say’ or ‘missing’ responses were treated as separate categories if they exceeded 5% of total responses to the question. To evaluate the robustness of multivariable models, a sensitivity analysis was conducted using backwards selection with Bayesian information criterion (BIC) to construct multivariable logistic regression models.

## Results

### Primary analysis: detailed descriptive statistics

The CASCARA patient survey had 606 responses. 470 (78%) and 136 (22%) participants reported experience of PBC and MBC, respectively. For PBC participants, 314 (67%) were aged 41–60 at diagnosis, 468 (99.6%) were female, 446 (95%) reported their ethnicity as White, 333 (71%) had a postgraduate degree/degree/professional qualification, and 373 (79%) were in a married/cohabiting relationship at diagnosis. For MBC participants, 99 (73%) were aged 41–60 at diagnosis, 136 (100%) were female, 135 (99%) reported their ethnicity as White, 84 (62%) had a postgraduate degree/degree/professional qualification, and 105 (77%) were in a married/cohabiting relationship at diagnosis. 27% of PBC participants (125/470) and 35% of MBC participants (48/136) reported experience of financial problems [14].

The CASCARA carer survey had 30 responses and therefore only descriptive statistics are reported in this manuscript, given the small number of responses. 17 (57%) carer participants were aged 41–60 when they started caring for someone with breast cancer, 18 (60%) were male, 29 (97%) reported their ethnicity as White, 19 (63%) had a postgraduate degree/degree/professional qualification and 25 (83%) were in a married/cohabiting relationship when they became carer. 21 (70%) carer participants were a partner to the patient and 17 (57%) cared for a patient with experience of MBC. Characteristics of all participants are displayed in Table 1.

**Table 1:** Baseline demographic and cancer-related characteristics.

		Participants with experience of PBC, *N* = 470		Participants with experience of MBC, *N* = 136		Carer, *N* = 30	
Cancer-related baseline characteristics (Carer provided cancer-related baseline characteristics of the patient they cared for.)							
Stage at most recent diagnosis		Primary with no recurrence	430 (91.5%)	Metastatic progression after first diagnosis	85 (62.5%)	Primary	12 (40.0%)
		Primary with local recurrence	18 (3.8%)	Metastatic since first diagnosed	50 (36.8%)	Metastatic	17 (56.7%)
		Primary with missing local recurrence information	22 (4.7%)	Metastatic with missing diagnosis time	1 (0.7%)	Not sure	1 (3.3%)
Time since diagnosis	Within the last year	129 (27.4%)		19 (14.0%)		4 (13.3%)	
	1 year ago	53 (11.3%)		14 (10.3%)		3 (10.0%)	
	2 years ago	73 (15.5%)		31 (22.8%)		7 (23.3%)	
	3 years ago	41 (8.7%)		18 (13.2%)		3 (10.0%)	
	4 years ago	35 (7.4%)		14 (10.3%)		5 (16.7%)	
	5 years ago	25 (5.3%)		5 (3.7%)		3 (10.0%)	
	More than 5 years ago	114 (24.3%)		35 (25.7%)		5 (16.7%)	
Treatment pathway	Awaiting first treatment	3 (0.6%)		0		0	
	Undergoing treatment at hospital	89 (18.9%)		106 (77.9%)		12 (40.0%)	
	Completed treatment at hospital and receiving ongoing treatment at home	241 (51.3%)		24 (17.6%)		8 (26.7%)	
	Completed all treatment	135 (28.7%)		4 (2.9%)		6 (20.0%)	
	Other	2 (0.4%)		1 (1.5%)		4 (13.3%)	
Region (s) receiving treatment	England	395 (84.0%)		89 (65.4%)		–	
	England, Scotland	2 (0.4%)		0		–	
	England, Wales	1 (0.2%)		1 (0.7%)		–	
	Northern Ireland	18 (3.8%)		24 (17.6%)		–	
	Scotland	37 (7.9%)		19 (14.0%)		–	
	Wales	17 (3.6%)		3 (2.2%)		–	
Demographics							
Age at diagnosis	<30	12 (2.6%)		0		3 (10.0%)	
	30–40	62 (13.2%)		16 (11.8%)		4 (13.3%)	
	41–50	155 (33.0%)		56 (41.2%)		2 (6.7%)	
	51–60	159 (33.8%)		43 (31.6%)		15 (50.0%)	
	61–70	70 (14.9%)		14 (10.3%)		4 (13.3%)	
	71–80	11 (2.3%)		6 (4.4%)		2 (6.7%)	
	>80	1 (0.2%)		1 (0.7%)		0	
Gender	Female	468 (99.6%)		136 (100.0%)		12 (40.0%)	
	Male	1 (0.2%)		0		18 (60.0%)	
	Other	1 (0.2%)		0		0	
Relationship to patient (only applicable to carer)	Partner	–		–		21 (70.0%)	
	Parent	–		–		3 (10.0%)	
	Child	–		–		1 (3.3%)	
	Sibling	–		–		2 (6.7%)	
	Other	–		–		3 (10.0%)	
Relationship status at diagnosis	Married/Cohabiting	373 (79.4%)		105 (77.2%)		25 (83.3%)	
	Single	55 (11.7%)		18 (13.2%)		5 (16.7%)	
	Divorced/Separated	28 (6.0%)		7 (5.1%)		0	
	Widowed	9 (1.9%)		5 (3.7%)		0	
	Prefer not to say	5 (1.1%)		1 (0.7%)		0	
Whether participants lived alone at diagnosis	No	405 (86.2%)		108 (79.4%)		29 (96.7%)	
	Yes	63 (13.4%)		27 (19.9%)		1 (3.3%)	
	Prefer not to say	2 (0.4%)		1 (0.7%)		0	
Ethnicity background	White	446 (94.9%)		135 (99.3%)		29 (96.7%)	
	Mixed or Multiple ethnic groups	5 (1.1%)		0		0	
	Asian or Asian British	5 (1.1%)		0		0	
	Black, Black British, Caribbean or African	5 (1.1%)		0		0	
	Other Ethnic Group	4 (0.9%)		0		0	
	Prefer not to say	5 (1.1%)		1 (0.7%)		1 (3.3%)	
Religion	No religion	192 (40.9%)		59 (43.4%)		13 (43.3%)	
	Christian	259 (55.1%)		70 (51.5%)		17 (56.7%)	
	Jewish	2 (0.4%)		0		0	
	Muslim	1 (0.2%)		0		0	
	Sikh	1 (0.2%)		0		0	
	Buddhist	0		1 (0.7%)		0	
	Other	3 (0.6%)		2 (1.5%)		0	
	Prefer not to say	12 (2.6%)		4 (2.9%)		0	
Education	Postgraduate degree/degree/professional qualification	333 (70.9%)		84 (61.8%)		19 (63.3%)	
	A level/HND or equivalent	69 (14.7%)		20 (14.7%)		4 (13.3%)	
	School certificate/GCSE/O level/NVQ or equivalent	60 (12.8%)		24 (17.6%)		7 (23.3%)	
	None	2 (0.4%)		7 (5.1%)		0	
	Prefer not to say	6 (1.3%)		1 (0.7%)		0	
Whether gender same as sex registered at birth	Yes	468 (99.6%)		134 (98.5%)		29 (96.7%)	
	No	1 (0.2%)		0		0	
	Prefer not to say	1 (0.2%)		2 (1.5%)		1 (3.3%)	
Sexual orientation	Straight/Heterosexual	440 (93.6%)		132 (97.1%)		27 (90.0%)	
	Gay/Lesbian	15 (3.2%)		2 (1.5%)		2 (6.7%)	
	Bisexual	5 (1.1%)		1 (0.7%)		0	
	Other	1 (0.2%)		0		0	
	Prefer not to say	9 (1.9%)		1 (0.7%)		1 (3.3%)	

#### Change in employment, income and unpaid caring responsibilities

For PBC participants, the most reported employment transition was moving from full-time to part-time employment. 28% (132/470) reported a >10% reduction in working hours and 8% (36/470) left employment, while those earning below the UK tax-free allowance (£12,570) rose from 15% (72/470) to 24% (114/470). Participants receiving benefits rose from 9% (40/470) to 14% (67/470). The most common benefits to receive were Personal Independence Payment (PIP) (*n* = 31) and Employment and Support Allowance (ESA) (*n* = 23). PIP is a UK benefit that helps with extra living costs for individuals who have difficulty with everyday tasks and/or problems getting around due to long-term health condition of disability. ESA provides financial support with living costs to people who are unable to work due to illness or disability, and may also offer help to return to work.

In contrast, for MBC participants, the most reported employment transition was retirement. 21% (28/136) reported a >10% reduction in working hours and 13% (18/136) left employment, while those earning below £12,570 rose from 17% (23/136) to 28% (38/136). Participants receiving benefits rose from 5% (7/136) to 46% (62/136). The most common benefits to receive were PIP (*n* = 50) and ESA (*n* = 28). Further details about employment for both patients and carers, including sick leave and sick pay, participants’ attitude towards change in employment are listed in Table 2 and Supplementary Tables A2–A8.

**Table 2:** Employment status, financial challenges and other difficulties for PBC participants, MBC participants and carer participants.

		Participants with experience of PBC, *N* = 470		Participants with experience of MBC, *N* = 136			Carer, *N* = 30	
		At diagnosis	At the time of survey completion	At diagnosis	At the time of survey completion	At diagnosis		At the time of survey completion
Work status	Full-time	252 (53.6%)	154 (32.8%)	79 (58.1%)	26 (19.1%)	16 (53.3%)		12 (40.0%)
	Part-time	109 (23.2%)	132 (28.1%)	29 (21.3%)	29 (21.3%)	4 (13.3%)		3 (10.0%)
	Not Paid & looking for employment	14 (3.0%)	13 (2.8%)	3 (2.2%)	2 (1.5%)	2 (6.7%)		5 (16.7%)
	Not Paid & Not looking for employment	31 (6.6%)	53 (11.3%)	7 (5.1%)	22 (16.2%)			
	Not Paid & Prefer not to say/Missing	1 (0.2%)	4 (0.8%)	0	1 (0.7%)			
	Retired	58 (12.3%)	109 (23.2%)	16 (11.8%)	52 (38.2%)	7 (23.3%)		9 (30.0%)
	Prefer not to say	4 (0.9%)	3 (0.6%)	2 (1.5%)	4 (2.9%)	1 (3.3%)		0
	Missing	1 (0.2%)	2 (0.4%)	0	0	0		1 (3.3%)
Working hours per week	Median [IQR]	37 [27.5–40]	30 [18–37]	37 [28.5–40]	24 [12–37]	37.5 [35.5–40]		37 [27–40]
Annual personal income pre-tax	<£12,570	72 (15.3%)	114 (24.3%)	23 (16.9%)	38 (27.9%)	4 (13.3%)		6 (20.0%)
	£12,570–25,000	102 (21.7%)	116 (24.7%)	32 (23.5%)	54 (39.7%)	9 (30.0%)		11 (36.7%)
	£25,001–50,000	175 (37.2%)	137 (29.1%)	49 (36.0%)	25 (18.4%)	10 (33.3%)		6 (20.0%)
	£50,001–75,000	53 (11.3%)	47 (10.0%)	20 (14.7%)	9 (6.6%)	3 (10.0%)		3 (10.0%)
	£75,001–100,000	22 (4.7%)	12 (2.6%)	4 (2.9%)	2 (1.5%)	1 (3.3%)		0
	>£100,000	15 (3.2%)	13 (2.8%)	3 (2.2%)	2 (1.5%)	1 (3.3%)		2 (6.7%)
	Prefer not to say	29 (6.2%)	28 (6.0%)	5 (3.7%)	6 (4.4%)	2 (6.7%)		2 (6.7%)
	Missing	2 (0.4%)	3 (0.6%)	0	0	0		0
Work schedule	Fixed working hours	192 (40.9%)	100 (21.3%)	54 (39.7%)	19 (14.0%)	11 (36.7%)		8 (26.7%)
	Flexible working hours	165 (35.1%)	176 (37.4%)	53 (39.0%)	32 (23.5%)	9 (30.0%)		7 (23.3%)
	NA	109 (23.2%)	184 (39.1%)	28 (20.6%)	81 (59.6%)	10 (33.3%)		15 (50.0%)
	Missing or Prefer not to say	4 (0.9%)	10 (2.2%)	1 (0.7%)	4 (3.0%)	0		0
Whether participants received benefits	Yes	29 (6.2%)	67 (14.3%)	7 (5.1%)	62 (45.6%)	2 (6.7%)		9 (30.0%)
	No	434 (92.3%)	394 (83.8%)	129 (94.9%)	71 (52.2%)	28 (93.3%)		21 (70.0%)
	Missing	7 (1.5%)	9 (1.9%)	0	3 (2.2%)	0		0
Whether participants were able to fulfil caring responsibilities	Time	Whilst undergoing treatment at hospital	At the time of survey completion	Whilst undergoing treatment at hospital	At the time of survey completion	Whilst the patient undergoing treatment at hospital		
	Not applicable	226 (48.1%)	237 (50.4%)	56 (41.2%)	60 (44.1%)	15 (50.0%)		–
	Yes	87 (18.5%)	193 (41.1%)	23 (16.9%)	39 (28.7%)	6 (20.0%)		–
	No	157 (33.4%)	39 (8.3%)	54 (39.7%)	35 (25.7%)	9 (30.0%)		–
	Missing	0	1 (0.2%)	3 (2.2%)	2 (1.5%)	0		–
Difficulty to cover costs associated with travel for treatment	Yes	369 (78.5)		100 (73.5%)			–	
	No	94 (20.0%)		34 (25.0%)			–	
	Not Applicable	1 (0.2%)		0			–	
	Missing	6 (1.3%)		2 (1.5%)			–	
Whether participants experienced financial problems due to additional costs or decrease in income following diagnosis of breast cancer	Yes	125 (26.6%)		48 (35.3%)			–	
	No	309 (65.7%)		70 (51.5%)			–	
	Prefer not to say	36 (7.7%)		18 (13.2%)			–	
More worries about money following diagnosis	Not at all	101 (21.7%)		13 (9.6%)			–	
	A little	196 (41.7%)		46 (33.8%)			–	
	Quite Often	90 (19.1%)		28 (20.6%)			–	
	Very Often	77 (16.4%)		49 (36.0%)			–	
	Prefer not to say	5 (1.1%)		0			–	
Need for financial support to cover costs associated with breast cancer	Yes	105 (22.3%)		45 (33.1%)			–	
	No	338 (71.9%)		73 (53.7%)			–	
	Prefer not to say	24 (5.1%)		18 (13.2%)			–	
	Missing	3 (0.6%)		0			–	
Whether participants had problems getting financial products	No	49 (10.4%)		10 (7.4%)			–	
	Yes	193 (41.1%)		86 (63.2%)			–	
	NA	221 (47.0%)		39 (28.7)			–	
	Prefer not to say	7 (1.5%)		1 (0.7%)			–	
Difficulties seeking help to manage physical or well-being needs related to breast cancer diagnosis	Yes	160 (34.0%)		63 (46.3%)			–	
	No	264 (56.2%)		63 (46.3%)			–	
	NA	44 (9.4%)		9 (6.6%)			–	
	Missing	2 (0.4%)		1 (0.7%)			–	

Among patient participants with caring responsibilities, a similar percentage of PBC (157/244, 64%) and MBC (54/77, 70%) participants reported difficulties fulfilling caring responsibilities at diagnosis, however more MBC participants continued to report difficulties at the time of survey completion (35/74, 47% MBC vs 39/242, 17% PBC) (Table 2). Unpaid care from partners, family member and friends were the most common support participants had at diagnosis (178/211, 84%) and at the time of survey completion (55/74, 74%).

#### Additional expenditure and financial issues

The most common additional expenditures due to breast cancer were “*clothing such as special bras or compression sleeves*” (339/470, 72%) and “*heating and fuel bills*” (116/136, 85%) for PBC and MBC participants, respectively (Supplementary Table A9). Additional expenditures on travel costs for treatment are listed in Table 3.

**Table 3:** Additional expenditures related to breast cancer.

	Participants with experience of PBC, *N* = 470	Participants with experience of MBC, *N* = 136
Additional health expenditures related to breast cancer in the last 6 months (Select all that applies)		
Prescription medicine	76 (16.2%)	11 (8.1%)
Dental visits	185 (39.4%)	91 (66.9%)
Supportive medication/equipment to treat side-effects (for example, moisturising creams, fan for hot flushes)	281 (59.8%)	93 (68.4%)
Dietary and other supplements	182 (38.7%)	67 (49.3%)
Herbal remedies	70 (14.9%)	29 (21.3%)
Complementary treatments	113 (24.0%)	56 (41.2%)
Sport and exercise	134 (28.5%)	41 (30.1%)
Private healthcare	46 (9.8%)	10 (7.4%)
Care provided at home	14 (3.0%)	6 (4.4%)
Supportive care (for example, massage, counselling, acupuncture, reflexology, yoga)	158 (33.6%)	67 (49.3%)
Wigs, hairpieces, head coverings	190 (40.4%)	56 (41.2%)
Clothing (for example, special bra, compression sleeves)	339 (72.1%)	79 (58.1%)
Modification of home (for example, installing handrails) or to your car	24 (5.1%)	17 (12.5%)
Other	16 (3.4%)	5 (3.7%)
None	55 (11.7%)	9 (6.6%)
Additional living expenditures due to breast cancer in the last month (Select all that applies)		
Heating and fuel bills	237 (50.4%)	116 (85.3%)
Food and drink	169 (36.0%)	69 (50.7%)
Travel costs	159 (33.8%)	79 (58.1%)
Household items	79 (16.8%)	37 (27.2%)
Telephone or internet bills	37 (7.9%)	21 (15.4%)
Personal care provided at home (for example, cleaning, cooking)	59 (12.6%)	25 (18.4%)
Childcare	17 (3.6%)	8 (5.9%)
New clothing for new body shape/needs	263 (56.0%)	83 (61.0%)
New makeup, eyebrow tinting, false lashes, and other personal care	169 (36.0%)	67 (49.3%)
Other	2 (0.4%)	2 (1.5%)
None	94 (20.0%)	6 (4.4%)

The most commonly reported financial problem was “*change of future plans*” (108/606, 18%), with participants elaborating in comments on changes such as “*altering retirement plans*”, “*being unable to afford any leisure activities*”, and “*falling into or worsening debts*”. Some participants also reported “*change or loss of home*”, “*unable to pay bills, mortgage or rent*” (Supplementary Table A10). 21% (127/606) participants discussed financial support with their breast cancer team and 14% (85/606) reported there was information available in hospital.

41% (193/470) PBC and 63% (86/136) MBC participants faced problems accessing financial products such as travel insurance, life and health insurance. Some participants also reported problems getting a mortgage or bank loan (Supplementary Table A11). 35% (163/470) PBC and 57% (77/136) MBC participants reported these problems were mainly related to their breast cancer diagnosis.

#### Physical, wellbeing issues, quality of life and access to research

99% (468/470) PBC and 100% (136/136) MBC participants reported some physical, or wellbeing issues related to breast cancer (Table 4). 34% (160/470) PBC and 46% (63/136) MBC participants reported difficulties seeking help to manage those issues (Table 2). Specifically, 62% (292/470) PBC and 68% (93/136) MBC of participants, reported impact on sexual health; 14% (67/470) PBC and 11% (15/136) MBC reported reduction in fertility. Participants mostly commonly sought help from their breast cancer team, general practitioners (GP), breast cancer support groups and charities (Supplementary Table A12). On a scale of 0–10 (10 = Meeting all needs, 0 = Meeting none of your needs), 14% (63/447) PBC and 20% (26/132) MBC participants rated support from NHS below or equal to 2. The median rating was 7 [Interquartile range 4–8] (PBC) and 6 [Interquartile range 4–8] (MBC). There was no statistically significant difference (Chi-square *p* = 0.7) between PBC and MBC participants in their ratings of NHS support, comparing those who rated it higher [5–10] versus lower (0–4). Among PBC participants, those diagnosed within the last two years (*p* < 0.001), currently undergoing hospital treatment (*p* = 0.003), or without financial problems (*p* = 0.005) were more likely to rate NHS support higher. Among MBC participants, those currently undergoing hospital treatment (Chi-square *p* = 0.047) were more likely to rate NHS support higher. 31% (190/606) and 16% (94/606) stated there should be more support on wellbeing or employment/finance for families and partners, respectively.

**Table 4:** Physical/wellbeing issues related to breast cancer.

	Participants with experience of PBC, *N* = 470			Participants with experience of MBC, *N* = 136		
	Total	Those with difficulty seeking help to manage their physical/wellbeing issues		Total	Those with difficulty seeking help to manage their physical/wellbeing issues	
		Frequency	% among those with this issue		Frequency	% among those with this issue
Lymphoedema	125 (26.6%)	56	44.8	40 (29.4%)	21	52.5
Menopausal symptoms (for example, hot flushes, joint aches/pains)	354 (75.3%)	135	38.1	106 (77.9%)	54	50.9
Impact on sexual health (for example, vaginal dryness, loss of sex drive)	292 (62.1%)	115	39.4	93 (68.4%)	45	48.4
Reduction in fertility	67 (14.3%)	29	43.3	15 (11.0%)	6	40.0
Anxiety	333 (70.9%)	132	39.6	90 (66.2%)	44	48.9
Depression	197 (41.9%)	104	52.8	61 (44.9%)	27	44.3
Memory problems	286 (60.9%)	123	43.0	96 (70.6%)	50	52.1
Loss of confidence	306 (65.1%)	126	41.2	90 (66.2%)	43	47.8
Concerns regarding body image	282 (60.0%)	118	41.8	82 (60.3%)	43	52.4
Pain	313 (66.6%)	130	41.5	95 (69.9%)	53	55.8
Nausea	167 (35.5%)	71	42.5	67 (49.3%)	38	56.7
Fatigue	390 (83.0%)	144	36.9	119 (87.5%)	61	51.3
Reduced mobility	182 (38.7%)	86	47.3	86 (63.2%)	46	53.5
Worsening of other medical conditions	111 (23.6%)	62	55.9	34 (25.0%)	24	70.6
Other	28 (6.0%)	16	57.1	5 (3.7%)	1	20.0
None	2 (0.4%)	–	–	0	–	–
Missing	2 (0.4%)	–	–	1 (0.7%)	–	–

**Fig. 1:**
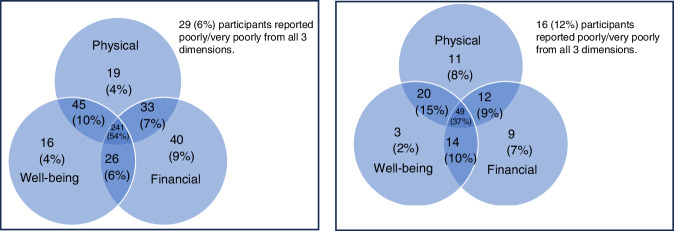
Quality of Life from physical, wellbeing and financial perspectives. Participants reported Well/Very Well quality of life from physical, wellbeing and financial perspectives for those with experience of primary breast cancer (left, *N* = 449) and metastatic breast cancer (right, *N* = 134).

54% (241/449) PBC and 37% (49/134) MBC participants rated ‘*Well*’ or ‘*Very Well*’ for QoL from a physical, wellbeing and financial perspective (Fig. 1). For all participants, physical and wellbeing QoL had moderate positive correlation (Spearman’s rank correlation ρ = 0.56 PBC, ρ = 0.43 MBC). For PBC participants only, their financial QoL had weak positive correlation with their physical (ρ = 0.39) and wellbeing QoL (ρ = 0.40). The impact of breast cancer on everyday activities is listed in Supplementary Table A13.

25% (118/470) PBC and 18% (25/136) MBC participants were offered opportunities to participate in clinical trials/research. Helping future patients was the most common reason cited for people to participate in research. Concern about unknown treatment benefit (PBC) and extra time commitment (MBC) was the most common reason for participants to decline research participation (Supplementary Table A14).

### Secondary analysis: relationship between factors

#### Physical QoL and wellbeing

Multivariable modelling shows that among PBC participants, lower education levels, prevalence of general disorders and administration site conditions (namely *pain, fatigue, reduced mobility or worsening of other medical conditions*), difficulty seeking help for physical/wellbeing issues, difficulty fulfilling caring responsibilities at the time of survey completion, were associated with worse physical QoL (Supplementary Table A15). For MBC participants, difficulty fulfilling caring responsibilities was found to be associated with worse physical QoL (Supplementary Table A16).

Among PBC participants, prevalence of any mental health and wellbeing issues (namely *anxiety, depression, memory problems, loss of confidence or concerns regarding body image*), difficulty seeking help for physical/wellbeing issues, difficulty fulfilling caring responsibilities, were associated with worse wellbeing QoL (Supplementary Table A17). Among MBC participants, lower education levels, difficulty seeking help for physical/wellbeing issues, difficulty fulfilling caring responsibilities, were associated with worse wellbeing QoL (Supplementary Table A18).

#### Financial QoL

Among PBC participants, lower income and experience of financial problems was associated with worse financial QoL (Supplementary Table A19). MBC participants with any change in employment (decreased hours, left employment, retired, or not in employment from the beginning) or experience of financial problems, reported worse financial QoL (Supplementary Table A20).

**Fig. 2:**
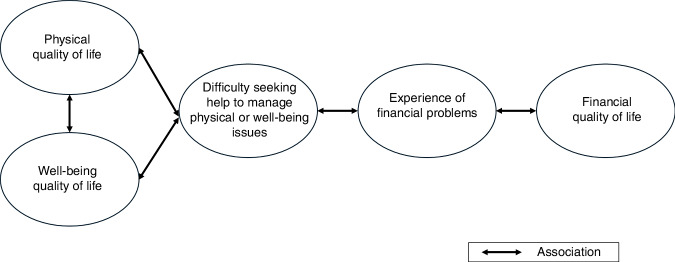
Association between physical, wellbeing and financial quality of life for participants with experience of PBC.

In exploratory analysis, difficulty seeking help for physical/wellbeing issues was significantly associated with experience of financial problems (PBC) or need for financial support (MBC). Factors associated with experience of financial problems included not being married, lower annual income, change of work status and others (Supplementary Table A21). Based on the observed associations, a conceptual model was constructed to potentially explain the relationship between physical, wellbeing QoL and financial QoL for participants with experience of PBC (Fig. 2).

## Discussion

This manuscript explored the relationships between physical, wellbeing, financial QoL and various factors, highlighting that experiences of people with PBC and MBC are distinct and require separate analysis. For PBC participants, multivariable regression analyses suggested that financial problems were associated with difficulty in accessing support for physical and wellbeing issues, which were associated with worse physical and wellbeing QoL. Relationship status, income, change in employment status and prevalence of physical or wellbeing issues were associated with financial problems. MBC Participants experience a complex interplay of factors affecting their QoL and financial problems. Hidden costs, financial problems and other challenges were more commonly reported than those with PBC, making it difficult to identify individual explanatory factors.

In published literature, the association between financial QoL and physical/wellbeing QoL is often attributed to delayed diagnosis, reduced, delayed or quitting treatment, or opting for less optimal treatment [2, 12, 13, 15]. However, our results showed that these factors do not fully account for the association as it persists even in a country where treatment is free via public healthcare. Supporting this, a study in the US by Wheeler et al. also showed that health insurance was a necessary but insufficient strategy to address cancer-related financial toxicities [16]. Our observation that the association between financial QoL and physical/wellbeing QoL was mediated by inadequate support of physical or wellbeing issues aligns with another study [17], which found participants with worse financial outcomes received worse quality of care, which was associated with worse QoL. In CASCARA, many participants also mentioned cancellations of vacations or no money for any leisure activities, which could be another possible explanatory factor. This echoes a finding from another US study among participants with experience of PBC showing “*sacrificing vacations and other ‘things’*” was associated with worse QoL [18].

Our analysis showed that financial problems were associated with sociodemographic and clinical factors for PBC participants. Financial problems were more common among those not in a married or cohabiting relationship [19–21], with lower income [20, 22–24], experienced change in employment status [19, 25] or faced physical/wellbeing issues, in agreement with other published research. Marriage may be associated with emotional or financial support [19] or better family resilience which helps with financial security [23]. In line with our findings, the published literature highlights other common factors affecting financial resilience such as younger age [16, 20, 21, 24, 26, 27], lower education level [12, 20], lower socioeconomic class [12, 27], self-employed [28, 29], higher grade of cancer [16, 20, 22], minority ethnicity group [24, 27].

Employment disruption, such as decreasing working hours, leaving employment, including early retirement [30, 31], was significantly associated with financial problems among people with experience of PBC in our study and aligned with other literature [19]. Physical issues like fatigue, lymphoedema, pain and arm motion limitation may explain some loss of productivity [32]. Mental health issues like loss of confidence, concern over body image, memory problems and cognitive problems may also be related to difficulty with returning to work [33–35]. Employment factors such as a non-supportive workplace, lack of sick leave, physical demands at work, little flexibility in working hours maybe barriers to return to work [36, 37]. Addressing these barriers by giving more relevant support and creating more supportive work environments may help reduce employment disruption. Returning to work is often seen as returning to usual life and may provide a sense of structure, self-worth, accomplishment and social connections [33].

Comparing with PBC participants, additional spending, difficulty seeking help, financial problems and other challenges were reported more by MBC participants. However, there were no specific explanatory factors identified for physical QoL or financial problems among MBC participants. This may be because all factors investigated were commonly reported and impacted on QoL and financial problems to a similar extent, so there was no evidence of the individual factors making a difference in regression models. For instance, 96% of MBC participants reported mental health issues, such as anxiety, and 92% reported general disorders, including pain and fatigue. The experiences of individuals with MBC appear to differ substantially from those with PBC experience, highlighting the need for further research specifically focused on MBC patients to better understand and support their unique needs.

Disruption to caring responsibilities was also commonly reported in our study, particularly among MBC participants. Although both PBC and MBC participants experienced this difficulty at diagnosis, 47% of MBC participants with caring responsibilities were still unable to fulfil them at the time of survey completion. In most cases, these responsibilities were taken on by partners, family, and friends.

Our study suggests more targeted support is needed to better address different needs for breast cancer patients. Long-term support for physical and wellbeing issues related to breast cancer should be available after active treatment ends and made accessible to patients and their family members. For example, sexual health is a vital component of overall quality of life, with poor sexual functioning linked to emotional distress, mental health challenges, and relationship difficulties [38]. Although commonly experienced by breast cancer patients, sexual health concerns are often overlooked in clinical care and can be difficult for clinicians to address in routine consultations [39]. Support for family and caregiving responsibilities can allow patients to focus on their own self-care. Information for financial and other support should be accessible during all stages of the disease. Appropriate government policies are needed to guide employers create more supportive workplaces. Health care professionals could also have a more active role in sign posting assistance with returning to work [33]. For example, in Germany, social service counselling to support cancer patients in reintegrating into everyday life demonstrated efficacy improving employment rate for patients [40]. Health systems need to be designed and implemented to address more holistic support addressing these far-reaching patient needs.

The relationships between physical, wellbeing and financial QoL suggest need for development of new measuring tools. Traditional health metrics have primarily focused on physical health outcomes, with some consideration given to mental wellbeing, and there is no standard tool and thus limited information about the important wider influence of breast cancer [41, 42]. The COmprehensive Score for financial Toxicity (COST) [43] is the most commonly used validated tool to describe financial distress experienced by cancer patients. It can provide a generic understanding of financial toxicities, but further tools are needed to provide more detailed understanding of other wider influences and causes of financial problems experienced by patients.

As part of a sensitivity analysis, the multivariable logistic regression model for QoL was re-analysed using backward selection based on BIC. The factors identified in the initial analysis were consistently recognised by this alternative method, indicating their robustness. However, the alternative approach also revealed several additional predictors, which raises concerns that the BIC may impose insufficient penalties during the model selection process and original method is preferred.

This study had several strengths. The questionnaire was developed in collaboration with volunteers with experience of breast cancer from diverse locations and socio-economic backgrounds across the UK. It covered a wide range of topics and aimed to provide a holistic view of the quality of life for people with experience of breast cancer. Using the format of an online survey, participants from diverse locations were able to participate. The same set of questions was answered by people with experience of PBC and MBC, making it possible to compare and contrast responses from each group.

Our limitations included our web-based disseminated model and short timeframe, introducing possible bias between whole UK breast cancer population and our study population. Access was therefore limited to those with access to internet which can exclude certain demographics (e.g. older, lower income adults). The participants appeared to report a higher education level, a higher annual income and were mostly White, where higher financial security would be expected. Specifically, 69% (435/636) held a postgraduate degree/degree/professional qualification, compared to 27% of the general British population aged 25-64 in 2023 [44]. 96% (610/636) identified as White, higher than 82% reported in 2021 England and Wales Census [45]. CASCARA also included a higher proportion of younger patients, with 15% (90/606) under 40, compared to just 4% of breast cancer patients under 40 in the UK [46]. This suggests that the wider influence of breast cancer is non-negligible and could be underestimated in this study. This study is cross-sectional and is limited to exploring associations rather than causation, as determining cause and effect requires longitudinal ascertainment. Due to the small sample size, only descriptive statistics were used for carer participants. Future research should aim to recruit more carers to better understand their experiences and perspectives.

## Conclusion

In conclusion, this study provided evidence for broader impact of breast cancer on people’s lives, including employment disruption, financial difficulties, overall QoL in a high-income country with a national health service. PBC participants with financial problems were more likely to experience difficulty seeking help for their physical/wellbeing issues, which links with worse physical and wellbeing QoL. More MBC participants experienced financial problems, unmet support needs and other issues related to breast cancer than PBC participants. With many commonly reported problems that may impact QoL to a similar extent, there was no evidence of specific factors influence QoL for MBC participants. Given that the CASCARA sample was predominantly white, married, and educated, future research should explore the financial impacts and unmet support needs in more diverse populations to ensure broader representation. We call for future clinical trials to include the wider impact of breast cancer as part of data collection. Better understanding of the wider impact may lead to better policy and support from healthcare professionals, employers and the wider society.

## Supplementary information

Appendix

Supplementary Material 1 CASCARA patient survey

Supplementary Material 2 CASCARA carer survey

## Data Availability

The data analysed in this study are securely managed by the clinical trials and statistics unit at the Institute of Cancer Research and are not available as part of a public database. The data can be available on request from the corresponding author.

## Code availability

All analyses were carried out using STATA/BE version 17. All data and code used as part of this manuscript will be available subject to ICR-CTSU data access policy.
