# Supplementary material for: Hidden costs and unmet supportive care needs among individuals with experience of breast cancer and their carers in the United Kingdom
Source: BJC Rep. 2025 Aug 19;3:55. doi: 10.1038/s44276-025-00172-z (PMC12361448; doi:10.1038/s44276-025-00172-z)
Supplement: Supplementary file 1 — Appendix [file 44276_2025_172_MOESM1_ESM.pdf]

APPENDIX

Table A1: Lists for endpoints and covariates

	Name	Corresponding questions
Primary endpoints	Physical QoL	Q51: How do you think you are doing at the moment 1) from a physical point of view? 2) from a well-being point of view? 3) from a financial point of view?<br>Answer: Very poorly/Poorly/Well/Very Well/Prefer not to say
	Wellbeing QoL	
	Financial QoL	
Exploratory endpoints	Experience of financial problems	Q29: Have you experienced any financial problems because of additional costs or a decrease in income?<br>Answer: Yes/No/Prefer not to say
	Need for financial support	Q32: Did you need any financial support to help cover costs associated with your breast cancer diagnosis?<br>Answer: Yes/No/Prefer not to say
	Difficulty to cover costs associated with travel for treatment	Q23-5/7 Was it difficult to cover the costs associated with travel for treatment after you received financial support?<br>Answer: Yes/No
	Caring responsibilities at the time of survey completion	Q19: Are you able to fulfil all your caring responsibilities now?<br>Answer: Not applicable/Yes/No
	Difficulty seeking help for physical/wellbeing issues	Q39: Did you have difficulties seeking help to manage the physical or well-being needs related to your breast cancer diagnosis?<br>Answer: Not applicable/Yes/No
Covariates	Age	Q55: What was your age at the time of your diagnosis?<br>Answer: Below 30 years/30-40 years/41-50 years/51-60 years/61-70 years/71-80 years/81 years and above/Prefer not to say

	Whether participants lived alone at the time of survey completion	Q61 Are you living alone now?<br>Answer: Yes/No/Prefer not to say
	Relationship status at the time of survey completion	Q60 What is your marital status now?<br>Answer: Single /”Married/Cohabiting”/”Divorced/Separated”/ Widowed / Prefer not to say<br>Divorced/Separated and widowed were merged in regression because of the low frequency in widowed category.
	Ethnicity	Q62: What is your ethnic group?<br>Answered: White/Mixed or Multiple ethnic groups/Asian or Asian British/Black, Black British, Caribbean or African, Other ethnic group/Prefer not to say<br>Mixed/Black/Asian/Other were merged in regression because of low frequency
	Education	Q64: Please select your highest education level:<br>Answer: Postgraduate degree/degree/professional qualification / A level/HND or equivalent / School certificate/GCSE/O-level/NVQ or equivalent / Other /None / Prefer not to say
	Stage of cancer	It was not be included as a factor in models, analysis was done separately for primary and metastatic participants.
	Time since diagnosis	Q2: When was your diagnosis of breast cancer?<br>Answer: Within the last 1 year/1 year ago/2 years ago/3 years ago/4 years ago/5 years ago/More than 5 years ago<br>This question is regrouped into within the last year, 1-5 years ago, more than 5 years ago to reduce too much noise.

	Treatment pathway	Q4: Where are you currently in the treatment pathway?<br>Answer: Awaiting your first treatment/Undergoing treatment at hospital/Completed treatment at hospital/Completed all treatment/Other<br>Awaiting first treatment and undergoing treatment at hospital were merged due to low frequency for those awaiting first treatment.
	Treatment location	Q6: Where did you receive your treatment?<br>Answer: England/Scotland/Wales/Northern Ireland
	Work status at diagnosis	Q7: What was your work status at the time you were diagnosed?<br>Answer: Full time employment/Part time employment/Not in paid employment/Retired/Prefer not to say
	Income at diagnosis	Q9: What was your estimated annual personal income pre-tax (including take home pay, benefits or any other regular income) at the time you were diagnosed?<br>Answer: Less than £12,570/£12,570-25,000/£25,001-50,000/£50,001-75,000/75,001-100,000/More than £100,000/Prefer not to say
	Change in work status	Defined from work status and hours: Increased hours (10% increase in average working hours per week), No change (Work) (Less than 10% change in average working hours per week), No change (Retired) (Work status at diagnosis and at the time of survey completion being both retirement), No change (Not paid) (Not in paid employment at diagnosis and at the time of survey completion), Decreased hours (10% decrease in average working hours per week), Retired (Work status at diagnosis being not retirement, work status at the time of survey completion being retirement), Quit employment (In full-time/part-time employment at diagnosis, not in paid employment at the time of survey completion), Unobtainable (Prefer not to say/Missing for work status or hours)

	Benefits at the time of survey completion	Q11: Are you or your partner receiving any benefits including universal credit **now**?<br>Answer: Yes/No/Prefer not to say
	Caring responsibilities at the time of survey completion	Q19: Are you able to fulfil all your caring responsibilities **now**?<br>Answer: Yes/No/Not applicable
	Experience of financial problems	Q29: Have you experienced any financial problems because of additional costs or a decrease in income?<br>Answer: Yes/No/Prefer not to say
	Need for financial support	Q32: Did you need any financial support to help cover costs associated with your breast cancer diagnosis?<br>Answer: Yes/No/Prefer not to say

	Physical or wellbeing issues related to breast cancer	Q36: Did you experience any of the following physical or well-being issues related to your breast cancer? (Select all that apply)<br>Answer: Lymphoedema/Menopausal symptoms/Impact on sexual health/Reduction in fertility/Anxiety/Depression/Memory problems/Loss of confidence/Concerns regarding body image/Pain/Nausea/Fatigue/Reduced mobility/Worsening of other medical conditions/Other/None<br><br>The physical/well-being issues are all created as binary variables. To reduce the number of factors within a model and also to reduce collinearity, the physical/well-being issues are grouped into more general classes. Medical Dictionary for Regulatory Activities Terminology (MedDRA) is referred to group the physical/well-being issues: Blood and lymphatic system disorders (Lymphoedema), Reproductive system and breast disorders (Menopausal symptoms, impact on sexual health, reduction in fertility), Mental health and wellbeing issues(Anxiety, depression, memory problems, loss of confidence, concerns regarding body image), General disorders and administration site conditions (Pain, fatigue, reduced mobility, worsening of other medical conditions), Gastrointestinal disorders (Nausea)
	Difficulty seeking help for physical/wellbeing issues	Q39: Did you have difficulties seeking help to manage the physical or well-being needs related to your breast cancer diagnosis?<br>Answer: Not applicable /Yes/No
The same set of covariates were evaluated for each of the endpoints except for the situation when the covariate and exploratory endpoints are the same.		

Table A2: Work Status of participants with experience of PBC at diagnosis and at the time of survey completion

	Work status at the time of survey completion	**Total**

Work status at diagnosis		Full-time	Part-time	Not Paid				Retired	Prefer not to say	Missing	
				Looking for employment	Not looking for employment	Prefer not to say	Missing				
Full-time		147(31.3%)	52(11.1%)	4(0.9%)	15(3.2%)	1(0.2%)		32(6.8%)		1(0.2%)	**252(53.6%)**
Part-time		4(0.9%)	75(16.0%)	5(1.1%)	11(2.3%)			14(3.0%)			**109(23.2%)**
Not Paid	Looking for employment	2(0.4%)	3(0.6%)	3(0.6%)	3(0.6%)			3(0.6%)			**14(3.0%)**
	Not looking for employment	1(0.2%)	2(0.4%)	1(0.2%)	24(5.1%)	1(0.2%)		2(0.4%)			**31(6.6%)**
	Prefer not to say					1(0.2%)					**1(0.2%)**
Retired								58(12.3%)			**58(12.3%)**
Prefer not to say									3(0.6%)	1(0.2%)	**4(0.9%)**
Missing							1(0.2%)				**1(0.2%)**
**Total**		**154(32.8%)**	**132(28.1%)**	**13(2.8%)**	**53(11.3%)**	**3(0.6%)**	**1(0.2%)**	**109(23.2%)**	**3(0.6%)**	**2(0.4%)**	**470(100.0%)**

Table A3: Annual personal income pre-tax of participants with experience of PBC at diagnosis and at the time of survey completion

Income at diagnosis	Income at the time of survey completion								**Total**
	<12,570	12,570-25,000	25,001-50,000	50,001-75,000	75,001-100,000	>100,000	Prefer not to say	Missing	
<12,570	60(12.8%)	9(1.9%)	2(0.4%)					1(0.2%)	**72(15.3%)**
12,570-25,000	24(5.1%)	66(14.0%)	10(2.1%)				2(0.4%)		**102(21.7%)**
25,001-50,000	21(4.5%)	37(7.9%)	106(22.6%)	10(2.1%)	1(0.2%)				**175(37.2%)**
50,001-75,000	2(0.4%)	3(0.6%)	13(2.8%)	32(6.8%)	1(0.2%)	1(0.2%)	1(0.2%)		**53(11.3%)**
75,001-100,000	3(0.6%)	1(0.2%)	5(1.1%)	4(0.9%)	9(1.9%)				**22(4.7%)**
>100,000	1(0.2%)		1(0.2%)		1(0.2%)	12(2.6%)			**15(3.2%)**
Prefer not to say	3(0.6%)			1(0.2%)			25(5.3%)		**29(6.2%)**
Missing								2(0.4%)	**2(0.4%)**
**Total**	**114(24.3%)**	**116(24.7%)**	**137(29.1%)**	**47(10.0%)**	**12(2.6%)**	**13(2.8%)**	**28(6.0%)**	**3(0.6%)**	**470(100.0%)**

Table A4: Benefits received by participants with experience of PBC at diagnosis and at the time of survey completion

Benefits at diagnosis	Benefits at the time of survey completion			**Total**
	No	Yes	Missing	
No	391(83.2%)	40(8.5%)	3(0.6%)	**434(92.3%)**
Yes	2(0.4%)	27(5.7%)		**29(6.2%)**
Missing	1(0.2%)		6(1.3%)	**7(1.5%)**
**Total**	**394(83.8%)**	**67(14.3%)**	**9(1.9%)**	**470(100.0%)**

Table A5: Sick Leave and attitude towards change in employment

		Participants with experience of PBC, N=470	Participants with experience of MBC, N=136	Carer, N=30
Did you take sick (or compassionate/carer's) leave?	Yes, it was sufficient to cover my needs	228(48.5%)	59(43.4%)	4(13.3%)
	Yes, it was not sufficient to cover my needs	74(15.7%)	28(20.6%)	4(13.3%)
	Yes (did not answer whether sufficient)	1(0.2%)	0	0
	No	41(8.7%)	13(9.6%)	12(40.0%)
	NA	124(26.4%)	35(25.7%)	10(33.3%)
	Missing	2(0.4%)	1(0.7%)	0
Did you receive sick pay during sick leave?/Did you receive any pay during compassionate/carer's leave?	During all my leave	206(43.8%)	60(44.1%)	7(23.3%)
	During part of my leave	75(16.0%)	22(16.2%)	0
	No pay	12(2.6%)	3(2.2%)	1(3.3%)
	Other	10(2.1%)	2(1.5%)	0
	NA	167(35.3%)	49(36.0%)	22(73.3%)
How much change in employment was influenced by breast cancer?	Not at all	57(12.1%)	10(7.4%)	4(13.3%)
	Slightly	61(13.0%)	7(5.1%)	3(10.0%)
	Somewhat	70(14.9%)	13(9.6%)	3(10.0%)
	Very much	123(26.2%)	73(53.7%)	8(26.7%)
	NA	155(33.0%)	32(23.5%)	12(40.0%)
	Missing	4(0.9%)	0	0
How do you feel about the changes in your employment since your (or the patient's)	Mostly happy	76(29.9%)	20(21.5%)	3(21.4)
	Somewhat happy	68(26.8%)	13(14.0%)	4(28.6%)
	Somewhat unhappy	61(24.0%)	31(33.3%)	4(28.6%)
	Mostly unhappy	46(18.1%)	29(31.2%)	3(21.4)

diagnosis? (Among participants who answered there was change in employment)	Missing	3(1.2%)	0	0
To what extent do you agree with the following statement: “I have been treated fairly at work after my breast cancer diagnosis (or since my caring role started).”	Strongly agree	159(33.8%)	43(31.6%)	8(26.7%)
	Agree	85(18.1%)	26(19.1%)	6(20.0%)
	Neutral	49(10.4%)	17(12.5%)	3(10.0%)
	Disagree	30(6.4%)	10(7.4%)	2(6.7%)
	Strongly disagree	19(4.0%)	9(6.6%)	3(10.0%)
	Prefer not to say	3(0.6%)	0	0
	NA	122(26.0%)	30(22.1%)	8(26.7%)
	Missing	3(0.6%)	1(0.7%)	0
To what extent do you agree with the following statement: 'I have been discriminated against at work due to my breast cancer diagnosis (or my caring role)'	Strongly agree	18(3.8%)	8(5.9%)	3(10.0%)
	Agree	31(6.6%)	8(5.9%)	2(6.7%)
	Neutral	53(11.3%)	19(14.0%)	4(13.3%)
	Disagree	82(17.4%)	17(12.5%)	4(13.3%)
	Strongly disagree	144(30.6%)	41(30.1%)	8(26.7%)
	Prefer not to say	4(0.9%)	1(0.7%)	0
	NA	136(28.9%)	41(30.1%)	9(30.0%)
	Missing	2(0.4%)	1(0.7%)	0

Table A6: Work Status of participants with experience of MBC at diagnosis and at the time of survey completion

Work status at diagnosis		Work status at the time of survey completion							Total
		Full-time	Part-time	Not Paid: Looking for employment	Not Paid: Not looking for employment	Not Paid: Missing	Retired	Prefer not to say	
Full-time		25(18.4%)	16(11.8%)	1(0.7%)	12(8.8%)	1(0.7%)	23(16.9%)	1(0.7%)	**79(58.1%)**
Part-time		1(0.7%)	12(8.8%)	1(0.7%)	3(2.2%)		11(8.1%)	1(0.7%)	**29(21.3%)**
Not Paid	Looking for employment		1(0.7%)		2(1.5%)				**3(2.2%)**
Not Paid	Not looking for employment				5(3.7%)		2(1.5%)		**7(5.1%)**
Retired							16(11.8%)		**16(11.8%)**
Prefer not to say								2(1.5%)	**2(1.5%)**
**Total**		**26(19.1%)**	**29(21.3%)**	**2(1.5%)**	**22(16.2%)**	**1(0.7%)**	**52(38.2%)**	**4(2.9%)**	**136(100.0%)**

Table A7: Annual personal income pre-tax of participants with experience of MBC at diagnosis and at the time of survey completion

Income at diagnosis	Income at the time of survey completion							**Total**
	<12,570	12,570-25,000	25,001-50,000	50,001-75,000	75,001-100,000	>100,000	Prefer not to say	
<12,570	15(11.0%)	7(5.1%)	1(0.7%)					**23(16.9%)**
12,570-25,000	8(5.9%)	24(17.6%)						**32(23.5%)**
25,001-50,000	12(8.8%)	16(11.8%)	19(14.0%)	1(0.7%)			1(0.7%)	**49(36.0%)**
50,001-75,000	2(1.5%)	7(5.1%)	3(2.2%)	8(5.9%)				**20(14.7%)**
75,001-100,000	1(0.7%)		2(1.5%)		1(0.7%)			**4(2.9%)**
>100,000					1(0.7%)	2(1.5%)		**3(2.2%)**
Prefer not to say							5(3.7%)	**5(3.7%)**
**Total**	**38(27.9%)**	**54(39.7%)**	**25(18.4%)**	**9(6.6%)**	**2(1.5%)**	**2(1.5%)**	**6(4.4%)**	**136(100.0%)**

Table A8: Benefits received by participants with experience of MBC at diagnosis and at the time of survey completion

Benefit at diagnosis	Benefit at the time of survey completion			**Total**
	No	Yes	Missing	
No	70(51.5%)	56(41.2%)	3(2.2%)	**129(94.9%)**
Yes	1(0.7%)	6(4.4%)		**7(5.1%)**
**Total**	**71(52.2%)**	**62(45.6%)**	**3(2.2%)**	**136(100.0%)**

Table A9: Travel costs related to treatment

		Participants with experience of PBC, N=470	Participants with experience of MBC, N=136
Average hospital visits per month during hospital-based treatment	Median [IQR]	5 [3 - 8]	4 [2 - 5]
Average travel costs per hospital visit (including parking and fuel)	Free	51(10.9%)	15(11.0%)
	Less than £10	276(58.7%)	82(60.3%)
	£10 - 30	121(25.7%)	36(26.5%)
	£31 - 50	15(3.2%)	0
	£51 - 100	4(0.9%)	3(2.2%)
	More than £100	2(0.4%)	0
	Missing	1(0.2%)	0
Furthest travel distance (one-way) for treatment at hospital	Less than 5 miles	94(20.0%)	25(18.4%)
	5-20 miles	215(45.7%)	65(47.8%)
	21-40 miles	118(25.1%)	31(22.8%)
	More than 40 miles	43(9.1%)	15(11.0%)
Ways to travel to hospital (Multiple choices)	On foot	38(8.1%)	5(3.7%)
	Bicycle	11(2.3%)	5(3.7%)
	Car driven by myself	242(51.5%)	90(66.2%)
	Car driven by unpaid drivers (e.g., friends, family or volunteers)	349(74.3%)	88(64.7%)
	Private car hire (e.g., taxi, Uber)	45(9.6%)	8(5.9%)
	Bus/Tram	51(10.9%)	14(10.3%)
	Train/Tube	43(9.1%)	11(8.1%)
	Hospital Transport	14(3.0%)	4(2.9%)
Costs related to Hospital visits (Multiple choices)	Travel cost excluding parking	402(85.5%)	122(89.7%)
	Parking	266(56.6%)	71(52.2%)
	Accommodation	24(5.1%)	2(1.5%)
	Miscellaneous costs	230(48.9%)	93(68.4%)

	Loss of earnings	81(17.2%)	20(14.7%)
	Childcare	0	0
	Other care services	5(1.1%)	4(2.9%)
	None	20(4.3%)	4(2.9%)
Spending per hospital visit (excluding loss of income)	Less than £10	278(59.1%)	84(61.8%)
	£10 - 30	153(32.6%)	45(33.1%)
	£31 - 50	17(3.6%)	3(2.2%)
	£51 - 100	12(2.6%)	2(1.5%)
	More than £100	6(1.3%)	1(0.7%)
	Missing	4(0.9%)	1(0.7%)

Table A10: Financial problems experienced by participants

	Participants with experience of PBC, N=470	Participants with experience of MBC, N=136
Loss of home	8(1.7%)	0
Change of home	5(1.1%)	2(1.5%)
Unable to pay bills	28(6.0%)	8(5.9%)
Not able to keep up mortgage/rent payments	15(3.2%)	2(1.5%)
Going into debt/worsening debt	57(12.1%)	16(11.8%)
Attending foodbanks	10(2.1%)	2(1.5%)
Missing treatments or doctor appointments	6(1.3%)	1(0.7%)
Change of future plans	80(17.0%)	28(20.6%)
Other financial problems	14(3.0%)	11(8.1%)
Prefer not to say	6(1.3%)	7(5.1%)

Table A11: Financial products that participants had problems getting

	Participants with experience of PBC		Participants with experience of MBC	
	No Problem	With Problem	No Problem	With Problem
Mortgage	9	18	2	9
Bank loan	4	8	0	2
Life Insurance	0	69	1	26
Health Insurance	0	56	1	15
Travel Insurance	37	159	6	80
Other	0	2	0	0

Table A12: Breast cancer support source

	Participants with experience of PBC, N=342	Participants with experience of MBC, N=109
The breast cancer team	237(69.3%)	69(63.3%)
General practitioner (GP)	200(58.5%)	68(62.4%)
Friends or family	135(39.5%)	39(35.8%)
Physiotherapy referred by NHS	69(20.2%)	23(21.1%)
Private physiotherapy	51(14.9%)	14(12.8%)
Information available from internet	107(31.3%)	36(33%)
Breast cancer support groups	167(48.8%)	70(64.2%)
Charity	157(45.9%)	54(49.5%)
Private healthcare	28(8.2%)	5(4.6%)
Private counsellor/psychologist	46(13.5%)	18(16.5%)
Social services/Council	0	0
Other	21(6.1%)	13(11.9%)
None	3(0.9%)	0

Table A13: How much breast cancer has impacted everyday activities on a scale of 0-10 (0=Very little and 10=Very much)

	Participants with experience of PBC, N=470					
Everyday activities \ Rating	0-3	4-6	7-10	Prefer not to say	NA	Missing
Caring	222(47.2%)	74(15.7%)	84(17.9%)		73(15.5%)	17(3.6%)
Employment	143(30.4%)	79(16.8%)	192(40.9%)		44(9.4%)	12(2.6%)
Domestic activities	173(36.8%)	141(30.0%)	145(30.9%)	1(0.2%)	2(0.4%)	8(1.7%)
Volunteering	193(41.1%)	49(10.4%)	102(21.7%)	6(1.3%)	105(22.3%)	15(3.2%)
Hobbies	165(35.1%)	135(28.7%)	145(30.9%)	2(0.4%)	8(1.7%)	15(3.2%)
Exercise	122(26.0%)	136(28.9%)	200(42.6%)	1(0.2%)	2(0.4%)	9(1.9%)
Holidays	147(31.3%)	101(21.5%)	202(43.0%)	1(0.2%)	10(2.1%)	9(1.9%)
Overall	119(25.3%)	140(29.8%)	185(39.4%)	1(0.2%)	3(0.6%)	22(4.7%)
	Participants with experience of MBC, N=136					
Everyday activities \ Rating	0-3	4-6	7-10	Prefer not to say	NA	Missing
Caring	37(27.2%)	36(26.5%)	44(32.4%)		17(12.5%)	2(1.5%)
Employment	25(18.4%)	14(10.3%)	82(60.3%)		13(9.6%)	2(1.5%)
Domestic activities	30(22.1%)	32(23.5%)	74(54.4%)			
Volunteering	39(28.7%)	12(8.8%)	44(32.4%)	1(0.7%)	35(25.7%)	5(3.7%)
Hobbies	29(21.3%)	37(27.2%)	64(47.1%)		4(2.9%)	2(1.5%)
Exercise	25(18.4%)	22(16.2%)	88(64.7%)		1(0.7%)	
Holidays	25(18.4%)	17(12.5%)	91(66.9%)		3(2.2%)	
Overall	17(12.5%)	21(15.4%)	97(71.3%)			1(0.7%)
	Carers, N=30					
Everyday activities \ Rating	0-3	4-6	7-10	Prefer not to say	NA	Missing
Caring	13(43.3%)	3(10.0%)	5(16.7%)		7(23.3%)	2(6.7%)
Employment	12(40.0%)	4(13.3%)	9(30.0%)		4(13.3%)	1(3.3%)
Domestic activities	10(33.3%)	4(13.3%)	15(50.0%)			1(3.3%)
Volunteering	11(36.7%)	2(6.7%)	6(20.0%)		10(33.3%)	1(3.3%)
Hobbies	11(36.7%)	4(13.3%)	13(43.3%)			2(6.7%)
Exercise	11(36.7%)	6(20.0%)	11(36.7%)		1(3.3%)	1(3.3%)
Holidays	10(33.3%)	3(10.0%)	16(53.3%)			1(3.3%)
Overall	7(23.3%)	8(26.7%)	13(43.3%)			2(6.7%)

Table A14: Reasons to accept or decline clinical trial/research

Reasons to accept clinical trial/research		
	Participants with experience of PBC, N=83	Participants with experience of MBC, N=15
To receive a new treatment	11(13.3%)	10(66.7%）
To potentially help live longer	26(31.3%)	10(66.7%）
To potentially improve quality of life	22(26.5%)	8(53.3%)
To receive closer monitoring/frequent follow-ups	37(44.6%)	11(73.3%)
To help breast cancer patients in the future	74(89.2%)	14(93.3%)
Other reasons	4(4.8%)	0
Reasons to decline clinical trial/research		
	Participants with experience of PBC, N=19	Participants with experience of MBC, N=7
Extra time commitment needed	5(26.3%)	4(57.1%)
Extra costs due to travel and hospital visits	3(15.8%)	3(42.9%)
Relationship with the clinical teams	1(5.3%)	1(14.3%)
Concern about possible side-effects	5(26.3%)	3(42.9%)
Concern that treatment was new so benefit not known	7(36.8%)	1(14.3%)
Concern about getting a placebo treatment	5(26.3%)	2(28.6%)
Additional information/paperwork seemed overwhelming	5(26.3%)	0
Concerns from family/friends	3(15.8%)	0
No specific factors	5(26.3%)	1(14.3%)
Other reasons	6(31.6%)	2(28.6%)
Other reasons to accept clinical trial: psychological support, genetic related test to help family. Other reasons to decline clinical trial: not available during specific dates, not wanting another biopsy, frightened, hard to find out side effects related to existing disability, delayed starting time.		

Table A15: Factors associated with physical QoL for PBC participants

Note: Greyed covariates were those that were not eligible for multivariate analysis. Bolded covariates were those that had a smaller p-value than the Benjamini-Hochberg threshold.

Factor		Number of responses	Univariable					Multivariable (N=441)				
			Number of observations	Odds ratio	Lower limit	Upper Limit	P-value	Odds ratio	Lower limit	Upper Limit	P-value	Benjamini-Hochberg
Age	<40	73	466	1.00	-	-	0.2					
	41-50	154		1.45	0.85	2.48						
	51-60	158		1.48	0.87	2.52						
	>60	81		1.90	1.03	3.51						
Whether lived alone at the time of survey completion	No	393	464	1.00	-	-	0.29					
	Yes	71		0.77	0.47	1.25						
Relationship Status at the time of survey completion	Married/Cohabiting	358	461	1.00	-	-	0.049	1.00	-	-	0.33	
	Single	56		0.50	0.29	0.87		0.65	0.35	1.24		
	Divorced/Separated/Widowed	47		0.94	0.53	1.67		1.18	0.61	2.28		
Ethnicity Background	White	442	461	1.00	-	-	0.19					
	Mixed/Asian/Black/Other	19		1.79	0.74	4.31						
**Education**	**Postgraduate degree/ degree/ professional qualification**	**331**	**460**	**1.00**	**-**	**-**	**0.01**	**1.00**	**-**	**-**	**0.008**	**0.017**
	**A level/HND or equivalent**	**68**		**0.56**	**0.34**	**0.93**		**0.59**	**0.34**	**1.02**		
	**School certificate/GCSE/O-level/ NVQ or equivalent or None**	**61**		**0.56**	**0.34**	**0.95**		**0.43**	**0.24**	**0.78**		
Time since diagnosis	Within last year	129	466	1.00	-	-	0.1					
	1 year ago	53		1.06	0.58	1.92						
	2 years ago	73		1.24	0.70	2.17						
	3 years ago	40		0.90	0.45	1.78						
	4 years ago	34		1.54	0.73	3.23						
	5 years ago	25		0.57	0.26	1.24						

	More than 5 years ago	112		1.70	1.04	2.79						
Treatment pathway	Awaiting your first treatment/Undergoing treatment at hospital	92	466	1.00	-	-	0.028	1.00	-	-	0.03	0.02
	Completed treatment at hospital	240		1.44	0.90	2.30		1.60	0.93	2.76		
	Completed all treatment/Other	134		2.02	1.20	3.40		2.27	1.25	4.14		
Treatment location	England	394	466	1.00	-	-	0.63					
	Scotland/Wales/Northern Ireland	72		1.12	0.70	1.80						
Work Status at diagnosis	Full-time	251	461	1.00	-	-	0.03	1.00	-	-	0.04	0.025
	Part-time	109		1.19	0.77	1.83		0.98	0.61	1.58		
	Not in paid employment	44		1.61	0.86	2.99		2.19	1.08	4.44		
	Retired	57		2.19	1.25	3.83		1.98	1.03	3.82		
Income at diagnosis	<£12,570	71	435	1.00	-	-	0.71					
	£12,570 - 25,000	101		1.34	0.75	2.41						
	£25,001 - 50,000	174		1.01	0.60	1.71						
	£50,001 - 75,000	52		1.34	0.67	2.68						
	>£75,000	37		1.09	0.51	2.33						
Change of work status	No change (Work)	126	461	1.00	-	-	0.03					
	Increased Hours	27		1.72	0.74	3.97						
	No change (Retire)	57		2.00	1.09	3.67						
	No change (Not paid)	33		1.10	0.52	1.33						
	Decreased Hours	132		0.83	0.52	1.33						
	Retired	50		1.63	0.85	3.10						
	Quit employment	36		0.71	0.36	1.42						
Benefits at the time of survey completion	No	390	457	1.00	-	-	0.001	1.00	-	-	0.58	
	Yes	67		0.44	0.27	0.73		0.85	0.47	1.53		
**Whether participants were able to fulfil caring responsibilities at the time of survey completion**	**No**	**39**	**465**	**1.00**	**-**	**-**	**0.0001**	**1.00**	**-**	**-**	**0.002**	**0.01**
	**Yes**	**191**		**4.38**	**2.24**	**8.56**		**3.61**	**1.70**	**7.67**		
	**NA**	**235**		**1.54**	**5.74**	**1.43**		**2.31**	**1.10**	**4.86**		

Financial problem	No	306	466	1.00	-	-	<0.0001	1.00	-	-	0.14	
	Prefer not to say	36		0.47	0.24	0.93		0.63	0.39	1.00		
	Yes	124		0.35	0.23	0.52		0.88	0.39	2.01		
Need for financial support	No	336	463	1.00	-	-	<0.0001					
	Yes	103		0.43	0.28	0.66						
	Prefer not to say	24		0.33	0.15	0.74						
Blood and lymphatic system disorders *	No	341	464	1.00	-	-	0.10					
	Yes	123		0.72	0.48	1.07						
Reproductive system and breast disorders*	No	75	464	1.00	-	-	0.001	1.00	-	-	0.7	
	Yes	389		0.45	0.28	0.72		0.89	0.50	1.59		
Mental health and wellbeing issues*	No	39	464	1.00	-	-	0.0003	1.00	-	-	0.29	
	Yes	425		0.31	0.16	0.58		0.66	0.31	1.42		
**General disorders and administration site conditions***	**No**	**39**	**464**	**1.00**	**-**	**-**	**<0.0001**	**1.00**	**-**	**-**	**<0.0001**	**0.004**
	**Yes**	**425**		**0.13**	**0.06**	**0.25**		**0.19**	**0.09**	**0.42**		
Gastrointestinal disorders*	No	297	464	1.00	-	-	0.002	1.00	-	-	0.51	
	Yes	167		0.56	0.39	0.81		0.87	0.57	1.32		
**Difficulty seeking help for physical/well-being issues**	**No**	**261**	**464**	**1.00**	**-**	**-**	**<0.0001**	**1.00**	**-**	**-**	**0.001**	**0.008**
	**Yes**	**159**		**0.42**	**0.29**	**0.62**		**0.53**	**0.35**	**0.81**		
	**NA**	**44**		**1.59**	**0.84**	**2.99**		**1.73**	**0.86**	**3.46**		

* The physical/well-being issues can be grouped according to MedDRA. A participant is considered to have a higher level AE, if the participant reported any AEs grouped under that higher level AE: Blood and lymphatic system disorders (Lymphoedema), Reproductive system and breast disorders (Menopausal symptoms, impact on sexual health, reduction in fertility), Mental health and wellbeing issues (Anxiety, depression, memory problems, loss of confidence, concerns regarding body image), General disorders and administration site conditions (Pain, fatigue, reduced mobility, worsening of other medical conditions), Gastrointestinal disorders (Nausea).

Table A16: Factors associated with physical QoL for MBC participants

Note: Greyed covariates were those that were not eligible for multivariate analysis. Bolded covariates were those that had a smaller p-value than the Benjamini-Hochberg threshold.

Factor		Number of responses	Univariable					Multivariable (N=125)				
			Number of observations	Odds ratio	Lower limit	Upper Limit	P-value	Odds ratio	Lower limit	Upper Limit	P-value	Benjamini-Hochberg
Age	31-40	16	136	1.00	-	-	0.3					
	41-50	56		1.26	0.45	3.53						
	51-60	43		1.25	0.43	3.62						
	>60	21		0.53	0.16	1.80						
Whether lived alone at the time of survey completion	No	110	135	1.00	-	-	0.2					
	Yes	25		1.69	0.74	3.87						
Relationship Status at the time of survey completion	Married/Cohabiting	107	135	1.00	-	-	0.11					
	Single	14		2.30	0.82	6.47						
	Divorced/Separated/Widowed	14		0.52	0.17	1.58						
Education	Postgraduate degree/ degree/ professional qualification	84	135	1.00	-	-	0.59					
	A level/HND or equivalent	20		0.64	0.25	1.65						
	School certificate/GCSE/O-level/ NVQ or equivalent	24		0.63	0.26	1.53						
	None	7		1.35	0.29	6.26						
Time since diagnosis	Within last year	19	136	1.00	-	-	0.18					
	1 year ago	14		4.37	1.17	16.29						
	2 years ago	31		1.24	0.42	3.66						
	3 years ago	18		0.94	0.28	3.16						
	4 years ago	14		1.60	0.43	5.95						
	More than 4 years ago	40		2.07	0.72	5.93						

Treatment pathway	Awaiting your first treatment/Undergoing treatment at hospital	106	136	1.00	-	-	0.68					
	Completed treatment at hospital/completed all treatment/Other	30		1.18	0.54	2.58						
Treatment location	England	89	136	1.00	-	-	0.77					
	Scotland/Wales/Northern Ireland	47		1.11	0.56	2.17						
Work Status at diagnosis	Full-time	79	134	1.00	-	-	0.42					
	Part-time	29		0.79	0.35	1.77						
	Not in paid employment	10		0.55	0.16	1.91						
	Retired	16		0.43	0.14	1.31						
Income at diagnosis	<£12,570	23	131	1.00	-	-	0.03	1.00	-	-	0.026	0.025
	£12,570 - 25,000	32		2.33	0.80	6.77		1.92	0.60	6.14		
	£25,001 - 50,000	49		2.86	1.09	7.51		1.72	0.53	5.57		
	£50,001 - 75,000	20		0.68	0.21	2.21		0.33	0.08	1.31		
	>£75,000	7		2.86	0.57	14.21		2.46	0.41	14.80		
Change of work status	No change (Work)/Increased hours	27	132	1.00	-	-	0.008	1.00	-	-	0.22	
	No change (Retire)	16		0.14	0.04	0.53		0.21	0.04	1.02		
	No change (Not paid)	7		0.11	0.02	0.60		0.22	0.03	1.56		
	Decreased Hours	28		0.21	0.07	0.58		0.38	0.11	1.25		
	Retired	36		0.33	0.12	0.89		0.71	0.22	2.28		
	Quit employment	18		0.17	0.05	0.56		0.34	0.09	1.28		
Benefits at the time of survey completion	No	71	133	1.00	-	-	0.09					
	Yes	62		0.57	0.29	1.10						
**Whether participants were able to fulfil caring responsibilitie**	**No**	**35**	**134**	**1.00**	**-**	**-**	**0.001**	**1.00**	**-**	**-**	**0.006**	**0.0125**
	**Yes**	**39**		**5.22**	**2.06**	**13.21**		**4.73**	**1.61**	**13.92**		
	**NA**	**60**		**3.30**	**1.44**	**7.60**		**4.11**	**1.53**	**11.04**		

**s at the time of survey completion**												
Financial problem	No	70	136	1.00	-	-	0.93					
	Yes	48		0.88	0.44	1.79						
	Prefer not to say	18		0.88	0.33	2.40						
Need for financial support	No	73	136	1.00	-	-	0.13					
	Yes	45		0.48	0.23	1.00						
	Prefer not to say	18		0.60	0.22	1.61						
Blood and lymphatic system disorders*	No	95	135	1.00	-	-	0.8					
	Yes	40		0.92	0.45	1.87						
Reproductive system and breast disorders*	No	20	135	1.00	-	-	0.73					
	Yes	115		1.17	0.47	2.93						
Mental health and wellbeing*	No	5	135	1.00	-	-	0.59					
	Yes	130		0.63	0.11	3.43						
General disorders and administration site conditions*	No	10	135	1.00	-	-	0.02	1.00	-	-	0.11	
	Yes	125		0.23	0.07	0.83		0.31	0.08	1.27		
Gastrointestinal disorders*	No	68	135	1.00	-	-	0.29					
	Yes	67		0.71	0.37	1.35						
Difficulty seeking help for physical/well-being issues	No	63	135	1.00	-	-	0.16					
	Yes	63		0.54	0.27	1.06						
	NA	9		1.15	0.30	4.42						

* The physical/well-being issues can be grouped according to MedDRA. A participant is considered to have a higher level AE, if the participant reported any AEs grouped under that higher level AE: Blood and lymphatic system disorders (Lymphoedema), Reproductive system and breast disorders (Menopausal symptoms, impact on sexual health, reduction in fertility), Mental health and wellbeing issues

(Anxiety, depression, memory problems, loss of confidence, concerns regarding body image), General disorders and administration site conditions (Pain, fatigue, reduced mobility, worsening of other medical conditions), Gastrointestinal disorders (Nausea).

Table A17: Factors associated with wellbeing QoL for PBC participants

Note: Greyed covariates were those that were not eligible for multivariate analysis. Bolded covariates were those that had a smaller p-value than the Benjamini-Hochberg threshold.

Factor		Number of responses	Univariable					Multivariable (N=444)				
			Number of observations	Odds ratio	Lower limit	Upper Limit	P-value	Odds ratio	Lower limit	Upper Limit	P-value	Benjamini-Hochberg
Age	<40	73	466	1.00	-	-	0.18					
	41-50	154		1.43	0.83	2.46						
	51-60	157		1.32	0.77	2.27						
	>60	82		1.98	1.07	3.68						
Whether lived alone at the time of survey completion	No	392	464	1.00	-	-	0.23					
	Yes	72		0.74	0.46	1.21						
Relationship Status at the time of survey completion	Married/Cohabiting	357	461	1.00	-	-	0.12					
	Single	56		0.57	0.33	0.98						
	Divorced/Separated/Widowed	48		0.97	0.54	1.73						
Ethnicity Background	White	442	461	1.00	-	-	0.76					
	Mixed/Asian/Black/Other	19		1.15	0.47	2.77						
Education	Postgraduate degree/ degree/ professional qualification	330	460	1.00	-	-	0.049	1.00	-	-	0.03	0.022
	A level/HND or equivalent	68		0.64	0.38	1.06		0.71	0.40	1.25		
	School certificate/GCSE/O-level/ NVQ or equivalent or None	62		0.58	0.34	0.99		0.45	0.25	0.84		
Time since diagnosis	Within last year	128	466	1.00	-	-	0.003					
	1 year ago	53		0.78	0.41	1.46						
	2 years ago	72		0.72	0.41	1.27						
	3 years ago	41		0.94	0.47	1.87						
	4 years ago	34		2.10	0.99	4.46						
	5 years ago	25		0.71	0.31	1.64						
	More than 5 years ago	113		2.02	1.21	3.36						

Treatment pathway	Awaiting your first treatment/Undergoing treatment at hospital	92	466	1.00	-	-	0.01	1.00	-	-	0.029	0.018
	Completed treatment at hospital	239		1.32	0.82	2.11		1.52	0.87	2.65		
	Completed all treatment/Other	135		2.16	1.27	3.66		2.28	1.23	4.23		
Treatment location	England	394	466	1.00	-	-	0.03	1.00	-	-	0.037	0.027
	Scotland/Wales/Northern Ireland	72		1.75	1.06	2.89		1.80	1.03	3.13		
Work Status at diagnosis	Full-time	249	461	1.00	-	-	0.02	1.00	-	-	0.21	
	Part-time	109		1.77	1.13	2.77		1.65	1.00	2.72		
	Not in paid employment	45		1.15	0.63	2.10		1.53	0.75	3.15		
	Retired	58		2.04	1.14	3.66		1.25	0.64	2.44		
Income at diagnosis	<£12,570	71	435	1.00	-	-	0.4					
	£12,570 - 25,000	102		1.56	0.85	2.87						
	£25,001 - 50,000	174		0.97	0.56	1.69						
	£50,001 - 75,000	52		1.15	0.56	2.38						
	>£75,000	36		1.09	0.50	2.39						
Change of work status	No change (Work)	125	461	1.00	-	-	0.08					
	Increased Hours	28		1.21	0.53	2.75						
	No change (Retire)	58		1.62	0.86	3.04						
	No change (Not paid)	33		1.00	0.48	2.05						
	Decreased Hours	131		0.81	0.50	1.32						
	Retired	50		1.53	0.79	2.96						
	Quit employment	36		0.51	0.24	1.06						
Benefits at the time of survey completion	No	390	457	1.00	-	-	0.002	1.00	-	-	0.51	
	Yes	67		0.46	0.28	0.76		0.82	0.44	1.50		
**Whether participants were able to fulfil caring responsibilities at the time of survey completion**	**No**	**39**	**465**	**1.00**	**-**	**-**	**0.0002**	**1.00**	-	-	**0.002**	**0.01**
	**Yes**	**191**		**4.03**	**2.02**	**8.04**		**3.70**	**1.65**	**8.29**		
	**NA**	**235**		**3.77**	**1.91**	**7.45**		**3.99**	**1.79**	**8.88**		

Financial problem	No	305	466	1.00	-	-	<0.0001	1.00	-	-	0.88	
	Prefer not to say	125		0.41	0.27	0.61		0.88	0.54	1.44		
	Yes	36		0.47	0.24	0.92		1.02	0.44	2.34		
Need for financial support	No	335	463	1.00	-	-	0.0001					
	Yes	104		0.43	0.28	0.66						
	Prefer not to say	24		0.38	0.17	0.84						
Blood and lymphatic system disorders*	No	341	464	1.00	-	-	0.04	1.00	-	-	0.99	
	Yes	123		0.66	0.44	0.98		1.00	0.62	1.60		
Reproductive system and breast disorders*	No	76	464	1.00	-	-	0.0002	1.00	-	-	0.41	
	Yes	388		0.40	0.24	0.65		0.78	0.43	1.41		
**Mental health and wellbeing issues***	**No**	**39**	**464**	**1.00**	**-**	**-**	**<0.0001**	**1.00**	-	-	**<0.0001**	**0.005**
	**Yes**	**425**		**0.11**	**0.06**	**0.21**		**0.14**	**0.07**	**0.32**		
General disorders and administration site conditions*	No	38	464	1.00	-	-	<0.0001	1.00	-	-	0.046	0.032
	Yes	426		0.20	0.10	0.38		0.45	0.21	0.99		
Gastrointestinal disorders*	No	297	464	1.00	-	-	0.0001	1.00	-	-	0.1	
	Yes	167		0.48	0.33	0.70		0.70	0.45	1.08		
**Difficulty seeking help for physical/well-being issues**	**No**	**263**	**464**	**1.00**	**-**	**-**	**<0.0001**	**1.00**	-	-	**<0.0001**	**0.005**
	**Yes**	**157**		**0.27**	**0.18**	**0.41**		**0.33**	**0.21**	**0.53**		
	**NA**	**44**		**1.56**	**0.80**	**3.04**		**1.53**	**0.74**	**3.15**		

* The physical/well-being issues can be grouped according to MedDRA. A participant is considered to have a higher level AE, if the participant reported any AEs grouped under that higher level AE: Blood and lymphatic system disorders (Lymphoedema), Reproductive system and breast disorders (Menopausal symptoms, impact on sexual health, reduction in fertility), Mental health and wellbeing issues (Anxiety, depression, memory problems, loss of confidence, concerns regarding body image), General disorders and administration site conditions (Pain, fatigue, reduced mobility, worsening of other medical conditions), Gastrointestinal disorders (Nausea).

Table A18: Factors associated with wellbeing QoL for MBC participants

Note: Greyed covariates were those that were not eligible for multivariate analysis. Bolded covariates were those that had a smaller p-value than the Benjamini-Hochberg threshold.

Factor		Number of responses	Univariable					Multivariable (N=132)				
			Number of observations	Odds ratio	Lower limit	Upper Limit	P-value	Odds ratio	Lower limit	Upper Limit	P-value	Benjamini-Hochberg
Age	31-40	16	136	1.00	-	-	0.67					
	41-50	56		1.73	0.59	5.05						
	51-60	43		1.62	0.53	4.91						
	>60	21		1.11	0.31	3.88						
Whether lived alone at the time of survey completion	No	110	135	1.00	-	-	0.15					
	Yes	25		1.87	0.79	4.38						
Relationship Status at the time of survey completion	Married/Cohabiting	107	135	1.00	-	-	0.29					
	Single	14		2.31	0.79	6.73						
	Divorced/Separated/Widowed	14		0.91	0.30	2.75						
**Education**	**Postgraduate degree/ degree/ professional qualification**	**84**	**135**	**1.00**	**-**	**-**	**0.03**	**1.00**	**-**	**-**	**0.034**	**0.05**
	**A level/HND or equivalent**	**20**		**0.23**	**0.08**	**0.64**		**0.20**	**0.06**	**0.63**		
	**School certificate/GCSE/O-level/ NVQ or equivalent**	**24**		**0.79**	**0.33**	**1.89**		**0.60**	**0.23**	**1.54**		
	**None**	**7**		**1.08**	**0.24**	**4.90**		**0.51**	**0.10**	**2.59**		
Time since diagnosis	Within last year	19	136	1.00	-	-	0.51					
	1 year ago	14		0.33	0.08	1.29						
	2 years ago	31		0.49	0.16	1.52						
	3 years ago	18		0.30	0.08	1.09						
	4 years ago	14		0.53	0.14	2.04						
	More than 4 years ago	40		0.42	0.14	1.26						

Treatment pathway	Awaiting your first treatment/Undergoing treatment at hospital	106	136	1.00	-	-	0.77					
	Completed treatment at hospital/completed all trt/Other	30		1.13	0.51	2.52						
Treatment location	England	89	136	1.00	-	-	0.43					
	Scotland/Wales/Northern Ireland	47		0.76	0.38	1.51						
Work Status at diagnosis	Full-time	79	134	1.00	-	-	0.11					
	Part-time	29		0.37	0.16	0.87						
	Not in paid employment	10		0.76	0.22	2.56						
	Retired	16		1.20	0.42	3.39						
Income at diagnosis	<£12,570	23	131	1.00	-	-	0.37					
	£12,570 - 25,000	32		1.57	0.56	4.39						
	£25,001 - 50,000	49		1.44	0.55	3.74						
	£50,001 - 75,000	20		1.12	0.34	3.73						
	>£75,000	7		4.96	1.00	24.56						
Change of work status	No change (Work)/Increased hours	27	132	1.00	-	-	0.17					
	No change (Retire)	16		0.77	0.23	2.58						
	No change (Not paid)	7		0.50	0.11	2.40						
	Decreased Hours	28		0.29	0.10	0.83						
	Retired	36		0.37	0.14	1.00						
	Quit employment	18		0.77	0.23	2.58						
Benefits at the time of survey completion	No	71	133	1.00	-	-	0.36					
	Yes	62		0.74	0.38	1.42						
**Whether participants were able to fulfil caring responsibilitie s at the time of**	**No**	**35**	**134**	**1.00**	**-**	**-**	**0.01**	**1.00**	**-**	**-**	**0.004**	**0.034**
	**Yes**	**39**		**3.04**	**1.22**	**7.56**		**4.13**	**1.52**	**11.26**		
	**NA**	**60**		**3.30**	**1.44**	**7.57**		**4.19**	**1.64**	**10.74**		

**survey completion**												
Financial problem	No	70	136	1.00	-	-	0.30					
	Yes	48		0.59	0.29	1.21						
	Prefer not to say	18		1.08	0.40	2.93						
Need for financial support	No	73	136	1.00	-	-	0.26					
	Yes	45		0.55	0.26	1.13						
	Prefer not to say	18		0.74	0.28	1.97						
Blood and lymphatic system disorders*	No	95	135	1.00	-	-	0.45					
	Yes	40		1.33	0.64	2.77						
Reproductive system and breast disorders*	No	20	135	1.00	-	-	0.78					
	Yes	115		1.13	0.46	2.80						
Mental health and wellbeing*	No	5	135	1.00	-	-	0.11					
	Yes	130		0.21	0.03	1.38						
General disorders and administration site conditions*	No	10	135	1.00	-	-	0.06					
	Yes	125		0.31	0.09	1.08						
Gastrointestinal disorders*	No	68	135	1.00	-	-	0.7					
	Yes	67		0.88	0.46	1.69						
**Difficulty seeking help for physical/well-being issues**	**No**	**63**	**135**	**1.00**	**-**	**-**	**0.0003**	**1.00**	**-**	**-**	**0.0001**	**0.017**
	**Yes**	**63**		**0.24**	**0.11**	**0.49**		**0.20**	**0.09**	**0.44**		
	**NA**	**9**		**0.78**	**0.19**	**3.09**		**1.02**	**0.25**	**4.21**		

* The physical/well-being issues can be grouped according to MedDRA. A participant is considered to have a higher level AE, if the participant reported any AEs grouped under that higher level AE: Blood and lymphatic system disorders (Lymphoedema), Reproductive system and breast disorders (Menopausal symptoms, impact on sexual health, reduction in fertility), Mental health and wellbeing issues (Anxiety, depression, memory problems, loss of confidence, concerns regarding body image), General disorders and administration site conditions (Pain, fatigue, reduced mobility, worsening of other medical conditions), Gastrointestinal disorders (Nausea).

Table A19: Factors associated with financial QoL for PBC participants

Note: Greyed covariates were those that were not eligible for multivariate analysis. Bolded covariates were those that had a smaller p-value than the Benjamini-Hochberg threshold.

Factor		Number of responses	Univariable					Multivariable (N=404)				
			Number of observations	Odds ratio	Lower limit	Upper Limit	P-value	Odds ratio	Lower limit	Upper Limit	P-value	Benjamini-Hochberg
Age	<40	72	453	1.00	-	-	0.01					
	41-50	152		0.85	0.49	1.46						
	51-60	150		0.97	0.56	1.67						
	>60	79		2.01	1.08	3.76						
Whether lived alone at the time of survey completion	No	384	451	1.00	-	-	0.003					
	Yes	67		0.46	0.28	0.76						
Relationship Status at the time of survey completion	Married/Cohabiting	351	449	1.00	-	-	<0.0001	1.00	-	-	0.12	
	Single	54		0.44	0.25	0.76		0.67	0.31	1.41		
	Divorced/Separated/ Widowed	44		0.29	0.16	0.55		0.43	0.18	1.04		
Ethnicity Background	White	430	449	1.00	-	-	0.99					
	Mixed/Asian/Black/Other	19		1.00	0.41	2.39						
Education	Postgraduate degree/ degree/ professional qualification	318	447	1.00	-	-	<0.0001	1.00	-	-	0.03	0.019
	A level/HND or equivalent	68		0.34	0.20	0.57		0.39	0.19	0.79		
	School certificate/GCSE/ O-level/ NVQ or equivalent or None	61		0.50	0.29	0.84		0.88	0.41	1.90		
Time since diagnosis	Within last year	124	453	1.00	-	-	0.15					
	1 year ago	53		0.70	0.38	1.30						

	2 years ago	69		0.88	0.50	1.56						
	3 years ago	40		0.81	0.39	1.68						
	4 years ago	34		1.58	0.76	3.28						
	5 years ago	23		0.48	0.21	1.12						
	More than 5 years ago	110		1.26	0.76	2.09						
Treatment pathway	Awaiting your first treatment/Undergoing treatment at hospital	87	453	1.00	-	-	0.14					
	Completed treatment at hospital	234		1.42	0.88	2.29						
	Completed all treatment/Other	132		1.70	1.00	2.90						
Treatment location	England	383	453	1.00	-	-	0.94					
	Scotland/Wales/Northern Ireland	70		0.98	0.60	1.60						
Work Status at diagnosis	Full-time	243	448	1.00	-	-	0.0002					
	Part-time	105		1.11	0.71	1.73						
	Not in paid employment	44		0.42	0.23	0.79						
	Retired	56		2.47	1.39	4.38						
**Income at diagnosis**	**<£12,570**	**71**	**424**	**1.00**	**-**	**-**	**<0.0001**	**1.00**	**-**	**-**	**0.005**	**0.008**
	**£12,570 - 25,000**	**94**		**2.32**	**1.26**	**4.28**		**2.82**	**1.26**	**6.34**		
	**£25,001 - 50,000**	**173**		**1.73**	**1.01**	**2.96**		**1.61**	**0.72**	**3.61**		
	**£50,001 - 75,000**	**49**		**5.33**	**2.57**	**11.05**		**4.66**	**1.74**	**12.52**		
	**>£75,000**	**37**		**3.69**	**1.70**	**8.01**		**2.32**	**0.80**	**6.77**		
Change of work status	No change (Work)	28	448	1.00	-	-	<0.0001	1.00	-	-	0.018	0.011
	Increased Hours	123		0.83	0.36	1.87		0.74	0.27	2.05		
	No change (Retire)	56		1.58	0.85	2.97		1.24	0.52	2.94		

	No change (Not paid)	32		0.19	0.09	0.42		0.46	0.13	1.65		
	Decreased Hours	127		0.44	0.27	0.73		0.56	0.31	1.03		
	Retired	48		1.17	0.61	2.26		0.86	0.40	1.84		
	Quit employment	34		0.11	0.05	0.24		0.17	0.06	0.48		
Benefits at the time of survey completion	No	380	444	1.00	-	-	<0.0001	1.00	-	-	0.34	
	Yes	64		0.21	0.12	0.35		0.69	0.32	1.48		
Whether participants were able to fulfil caring responsibilities at the time of survey completion	No	39	452	1.00	-	-	0.001	1.00	-	-	0.19	
	Yes	187		3.16	1.62	6.17		2.17	0.87	5.38		
	NA	226		3.28	1.69	6.35		2.26	0.91	5.61		
**Financial problem**	**No**	**303**	**453**	**1.00**	**-**	**-**	**<0.0001**	**1.00**	**-**	**-**	**<0.0001**	**0.004**
	**Prefer not to say**	**119**		**0.04**	**0.02**	**0.06**		**0.05**	**0.03**	**0.10**		
	**Yes**	**31**		**0.11**	**0.05**	**0.24**		**0.25**	**0.08**	**0.76**		
Need for financial support	No	328	450	1.00	-	-	<0.0001					
	Yes	101		0.07	0.04	0.12						
	Prefer not to say	21		0.10	0.04	0.25						
Blood and lymphatic system disorders*	No	333	451	1.00	-	-	0.003	1.00	-	-	0.23	
	Yes	118		0.54	0.35	0.81		1.38	0.82	2.32		
Reproductive system and breast disorders*	No	72	451	1.00	-	-	0.004	1.00	-	-	0.53	
	Yes	379		0.48	0.29	0.79		0.80	0.40	1.61		
Mental health and wellbeing*	No	36	451	1.00	-	-	<0.0001	1.00	-	-	0.026	0.015
	Yes	415		0.16	0.08	0.32		0.37	0.15	0.89		
General disorders and administration site conditions*	No	39	451	1.00	-	-	<0.0001	1.00	-	-	0.29	
	Yes	412		0.24	0.13	0.46		0.62	0.26	1.49		
Gastrointestinal disorders*	No	286	451	1.00	-	-	0.0004	1.00	-	-	0.18	
	Yes	165		0.51	0.35	0.74		0.72	0.45	1.17		
	No	254	452	1.00	-	-		1.00	-	-	0.36	

Difficulty seeking help for physical/well-being issues*	Yes	156		0.41	0.28	0.62	<0.0001	0.80	0.49	1.32		
	NA	42		1.59	0.85	2.97		1.45	0.67	3.14		

* The physical/well-being issues can be grouped according to MedDRA. A participant is considered to have a higher level AE, if the participant reported any AEs grouped under that higher level AE: Blood and lymphatic system disorders (Lymphoedema), Reproductive system and breast disorders (Menopausal symptoms, impact on sexual health, reduction in fertility), Mental health and wellbeing issues (Anxiety, depression, memory problems, loss of confidence, concerns regarding body image), General disorders and administration site conditions (Pain, fatigue, reduced mobility, worsening of other medical conditions), Gastrointestinal disorders (Nausea).

Table A20: Factors associated with financial QoL for MBC participants

Note: Greyed covariates were those that were not eligible for multivariate analysis. Bolded covariates were those that had a smaller p-value than the Benjamini-Hochberg threshold.

Factor		Number of responses	Number of observations	Univariable				Multivariable (N=129)				
				Odds ratio	Lower limit	Upper Limit	P-value	Odds ratio	Lower limit	Upper Limit	P-value	Benjamini-Hochberg
Age	31-40	16	134	1.00	-	-	0.11					
	41-50	55		2.39	0.76	7.50						
	51-60	42		3.32	1.01	10.95						
	>60	21		4.75	1.23	18.36						
Whether lived alone at the time of survey completion	No	108	133	1.00	-	-	0.67					
	Yes	25		0.83	0.36	1.94						
Relationship Status at the time of survey completion	Married/Cohabiting	105	133	1.00	-	-	0.3					
	Single	14		0.75	0.26	2.17						
	Divorced/Separated/ Widowed	14		2.31	0.69	7.72						
Education	Postgraduate degree/ degree/ professional qualification	83	133	1.00	-	-	0.53					
	A level/HND or equivalent	20		0.84	0.31	2.23						
	School certificate/GCSE/ O-level/ NVQ or equivalent	24		1.12	0.46	2.73						
	None	6		3.04	0.62	14.97						

Time since diagnosis	Within last year	19	134	1.00	-	-	0.13					
	1 year ago	13		0.52	0.13	2.12						
	2 years ago	31		0.49	0.15	1.61						
	3 years ago	18		0.24	0.06	0.89						
	4 years ago	14		1.15	0.28	4.70						
	More than 4 years ago	39		0.34	0.11	1.04						
Treatment pathway	Awaiting your first treatment/ Undergoing treatment at hospital	104	134	1.00	-	-	0.14					
	Completed treatment at hospital/ completed all trt/Other	30		1.85	0.81	4.23						
Treatment location	England	87	134	1.00	-	-	0.42					
	Scotland/Wales/Northern Ireland	47		1.33	0.66	2.69						
Work Status at diagnosis	Full-time	77	132	1.00	-	-	0.06					
	Part-time	29		0.87	0.37	2.01						
	Not in paid employment	10		0.84	0.24	3.02						
	Retired	16		4.19	1.33	13.21						
Income at diagnosis	<£12,570	23	129	1.00	-	-	0.58					
	£12,570 - 25,000	31		1.18	0.41	3.40						
	£25,001 - 50,000	48		0.73	0.28	1.93						
	£50,001 - 75,000	20		1.42	0.41	4.91						
	>£75,000	7		2.07	0.39	11.06						
**Change of work status**	**No change (Work)/Increased hours**	**26**	**130**	**1.00**	**-**	**-**	**<0.0001**	**1.00**	**-**	**-**	**0.0004**	**0.034**
	**No change (Retire)**	**16**		**0.93**	**0.23**	**3.74**		**0.59**	**0.13**	**2.59**		

	**No change (Not paid)**	**7**		**0.13**	**0.02**	**0.75**		**0.02**	**0.00**	**0.22**		
	**Decreased Hours**	**28**		**0.06**	**0.02**	**0.23**		**0.06**	**0.01**	**0.29**		
	**Retired**	**36**		**0.17**	**0.05**	**0.57**		**0.20**	**0.05**	**0.79**		
	**Quit employment**	**17**		**0.08**	**0.02**	**0.32**		**0.09**	**0.01**	**0.49**		
Benefits at the time of survey completion	No	70	131	1.00	-	-	0.22					
	Yes	61		0.65	0.33	1.29						
Whether participants were able to fulfil caring responsibilities at the time of survey completion	No	35	132	1.00	-	-	0.33					
	Yes	38		1.55	0.63	3.81						
	NA	59		1.86	0.82	4.25						
**Financial problem**	**No**	**69**	**134**	**1.00**	**-**	**-**	**<0.0001**	**1.00**	**-**	**-**	**<0.0001**	**0.017**
	**Yes**	**48**		**0.03**	**0.01**	**0.08**		**0.02**	**0.01**	**0.08**		
	**Prefer not to say**	**17**		**0.32**	**0.10**	**1.06**		**0.36**	**0.09**	**1.51**		
Need for financial support	No	73	134	1.00	-	-	<0.0001					
	Yes	45		0.16	0.07	0.36						
	Prefer not to say	16		0.51	0.17	1.50						
Blood and lymphatic system disorders*	No	93	133	1.00	-	-	0.62					
	Yes	40		0.83	0.40	1.72						
Reproductive system and breast disorders*	No	19	133				0.056					
	Yes	114		0.37	0.13	1.05						
Mental health and wellbeing*	No	5	133				0.6					
	Yes	128		0.64	0.11	3.52						
	No	10	133				0.07					

General disorders and administration site conditions*	Yes	123		0.31	0.08	1.15						
Gastrointestinal disorders*	No	68	133	1.00	-	-	0.18					
	Yes	65		0.63	0.32	1.24						
**Difficulty seeking help for physical/well-being issues**	**No**	**63**	**133**	**1.00**	**-**	**-**	**0.005**	**1.00**	**-**	**-**	**0.03**	**0.05**
	**Yes**	**62**		**0.32**	**0.16**	**0.67**		**0.28**	**0.10**	**0.76**		
	**NA**	**8**		**1.20**	**0.28**	**5.12**		**0.89**	**0.15**	**5.49**		

* The physical/well-being issues can be grouped according to MedDRA. A participant is considered to have a higher level AE, if the participant reported any AEs grouped under that higher level AE: Blood and lymphatic system disorders (Lymphoedema), Reproductive system and breast disorders (Menopausal symptoms, impact on sexual health, reduction in fertility), Mental health and wellbeing issues (Anxiety, depression, memory problems, loss of confidence, concerns regarding body image), General disorders and administration site conditions (Pain, fatigue, reduced mobility, worsening of other medical conditions), Gastrointestinal disorders (Nausea).

Table A21: Significant factors for exploratory endpoints

Endpoints / Factors	Participants with experience of PBC	Participants with experience of MBC
Experience of financial problems	Relationship status (single, divorced/separated/widowed), Annual income at diagnosis (<£12,570), change of work status (quit employment, decreased hours), prevalence of blood and lymphatic system disorders (Lymphoedema), mental health and wellbeing issues (Anxiety, depression, memory problems, loss of confidence, concerns regarding body image), general disorders and administration site conditions (Pain, fatigue, reduced mobility, worsening of other medical conditions), difficulty seeking help for physical/well-being issues	None
Need for financial support	Relationship status (divorced/separated/widowed), participants not able to fulfil caring responsibilities at the time of survey completion, prevalence of gastrointestinal disorders (nausea), difficulty seeking help for physical/well-being issues	Prevalence of blood and lymphatic system disorders (Lymphoedema), difficulty seeking help for physical/well-being issues
Difficulty to cover costs associated with travel for treatment	Education (A level or equivalent, School certificate or equivalent), difficulty seeking help for physical/well-being issues	Difficulty seeking help for physical/well-being issues
Ability to fulfil caring responsibilities at the time of survey completion	None	Analysis not done due to small number of participants with caring responsibilities
Difficulty seeking help for physical/well-being issues	Experience of financial problems	Need for financial support, prevalence of gastrointestinal disorders (nausea)
