# Supplementary material for: Hidden costs and unmet supportive care needs among individuals with experience of breast cancer and their carers in the United Kingdom
Source: BJC Rep. 2025 Aug 19;3:55. doi: 10.1038/s44276-025-00172-z (PMC12361448; doi:10.1038/s44276-025-00172-z)
Supplement: Supplementary file 2 — Supplementary Material 1 CASCARA patient survey [file 44276_2025_172_MOESM2_ESM.pdf]

CASCARA (Costs And Supportive Care in bReast cAncer) survey (for patients with breast cancer)

Introduction

The CASCARA research team are investigating the hidden financial costs and supportive care needs associated with breast cancer. This work is part of the Lancet Breast Cancer Commission which is looking at worldwide problems in breast cancer and how we can address them. We hope that by raising awareness of these hidden costs and supportive care needs, we can drive change towards better support for people with breast cancer and their families.

In this survey we would like to hear from people who have had a diagnosis of breast cancer and received their treatment in the UK. The survey should take about 20 minutes to complete. Your answers will be completely anonymous. Thank you so much for taking the time to complete this survey – your views are important and may help others in the future.

You can review our GDPR privacy statement on our website: https://www.icr.ac.uk/legal/privacy

If you have any questions about this research, please feel free to email any questions to the CASCARA team: **cascara-icrctsu@icr.ac.uk**

If you would be happy to take part, please click **Next** to continue.

Section 1: We would like to ask about your diagnosis of breast cancer.

If you have had more than one diagnosis of breast cancer or your cancer has returned, please provide information related to your most recent experience.

Q1 When you were most recently treated for breast cancer, was it for: (Required question)

- Primary (early) breast cancer (where cancer is only in the breast and/or lymph nodes) [show Q1-1]
- Secondary (metastatic) breast cancer (where breast cancer has spread to other parts of the body) [show Q1-2]

Q1-1 After you completed your treatment for primary (early) breast cancer, has your cancer come back in your breast or lymph nodes?

- Yes
- No

Q1-2 Which of the following options best describes how you were diagnosed with secondary (metastatic) breast cancer?

- The cancer had spread to other parts of your body when you were first diagnosed
- The cancer spread to other parts of your body sometime after you were first diagnosed

Q2 When was your diagnosis of breast cancer? (Required question)

- Within the last 1 year
- 1 year ago
- 2 years ago
- 3 years ago
- 4 years ago
- 5 years ago
- More than 5 years ago

Q3 Which of the following treatments have you received/are you planning to receive? (Select all that apply)

- Surgery
- Radiotherapy
- Chemotherapy (for example, EC-T)
- Targeted therapy (for example, trastuzumab, palbociclib, TDM-1)
- Endocrine therapy (for example, letrozole, tamoxifen)
- Ovarian suppression (for example, goserelin)
- Bisphosphonate-type drug (for example, zoledronic acid, denosumab)
- Immunotherapy (for example, atezolizumab, pembrolizumab)
- Not sure
- Other: free text box
- None

Q4 Where are you currently in the treatment pathway? (Required question)

- Awaiting your first treatment
- Undergoing treatment at hospital (for example, surgery, radiotherapy, chemotherapy, targeted therapy)
- Completed treatment at hospital (for example, surgery, radiotherapy, chemotherapy, targeted therapy) and receiving ongoing treatment at home (for example, endocrine therapy, ovarian suppression, bisphosphonates)
- Completed all treatment
- Other: free text box

Q5 Did you receive your diagnosis/treatment through: (Required question)

- National Health Service (NHS)
- Private health care [show Q5-1]
- A mixture of NHS and private health care [show Q5-1]

Q5-1 What are your reasons for using private health care? (Select all that apply)

- To access diagnosis/treatment more quickly
- To access treatment unavailable on the NHS
- To use private health insurance
- Other: free text box

Q6 Where did you receive your treatment? (Select all that apply) (Required question)

- England
- Scotland
- Wales
- Northern Ireland

Section 2: We would like to find out more about your employment and the impact of your breast cancer diagnosis on employment.

Q7 What was your work status **at the time you were diagnosed**?

- Full time employment (may include paid sick leave) [show Q7-2][show Q7-3][show Q7-4]
- Part time employment (may include paid sick leave) [show Q7-2][show Q7-3][show Q7-4]
- Not in paid employment [show Q7-1]
- Retired
- Prefer not to say

Q7-1 Were you looking for employment **at the time you were diagnosed**?

- Yes
- No
- Prefer not to say

Q7-2 On average, how many hours did you work per week **at the time you were diagnosed**? Please enter a whole number.

Free text box

Q7-3 How would you describe your work schedule **at the time you were diagnosed**?

- Fixed working hours (not able to adjust start and finish times)
- Flexible working hours (able to adjust start and finish times)
- Prefer not to say

Q7-4 Were you self-employed **at the time you were diagnosed**?

- Yes
- No
- Prefer not to say

Q8 Were you or your partner receiving any benefits including universal credit **at the time you were diagnosed**?

- Yes [show Q8-1]
- No
- Prefer not to say

Q8-1 If you or your partner were receiving any benefits **at the time you were diagnosed**, please can you tell us what benefits you were receiving?

Free text box

Q9 What was your estimated annual personal income pre-tax (including take home pay, benefits or any other regular income) **at the time you were diagnosed**?

- Less than £12,570
- £12,570-25,000
- £25,001-50,000
- £50,001-75,000
- £75,001-100,000
- More than £100,000
- Prefer not to say

Q10 What is your work status **now**?

- Full time employment (may include paid sick leave) [show Q10-2][show Q10-3][show Q10-4]
- Part time employment (may include paid sick leave) [show Q10-2][show Q10-3][show Q10-4]
- Not in paid employment [show Q10-1]
- Retired
- Prefer not to say

Q10-1 Are you looking for employment **now**?

- Yes
- No
- Prefer not to say

Q10-2 On average, how many hours do you work per week **now**? Please enter a whole number.

Free text box

Q10-3 How would you describe your work schedule **now**?

- Fixed working hours (not able to adjust start and finish times)
- Flexible working hours (able to adjust start and finish times)
- Prefer not to say

Q10-4 Are you self-employed **now**?

- Yes
- No
- Prefer not to say

Q11 Are you or your partner receiving any benefits including universal credit **now**?

- Yes [show Q11-1]
- No
- Prefer not to say

Q11-1 If you or your partner are receiving any benefits **now**, please can you tell us what benefits you receive?

Free text box

Q12 What is your estimated annual personal income pre-tax (including take home pay, benefits or any other regular income) **now**?

- Less than £12,570
- £12,570-25,000
- £25,001-50,000
- £50,001-75,000
- £75,001-100,000
- More than £100,000
- Prefer not to say

Q13 If there have been changes in your employment since your diagnosis, how much was this influenced by your breast cancer?

- Not applicable
- Not at all
- Slightly [Show Q13-1][Show Q13-2]
- Somewhat [Show Q13-1][Show Q13-2]
- Very much [Show Q13-1][Show Q13-2]

Q13-1 Overall, how do you feel about the changes in your employment since your diagnosis?

- Mostly happy
- Somewhat happy
- Somewhat unhappy
- Mostly unhappy

Q13-2 Please can you tell us how your diagnosis influenced your employment?

Free text box

Q14 To what extent do you agree with the following statement: “I have been treated fairly at work after my breast cancer diagnosis.”

- Not applicable
- Strongly agree
- Agree
- Neutral
- Disagree
- Strongly disagree
- Prefer not to say

Q15 To what extent do you agree with the following statement: “I have been discriminated against at work due to my breast cancer diagnosis.”

- Not applicable
- Strongly agree
- Agree
- Neutral
- Disagree
- Strongly disagree

- Prefer not to say

Q16 Did you take sick leave?

- Not applicable (for example, not in paid employment)
- Yes [show Q16-1][show Q16-2]
- No [show Q16-3]

Q16-1 Was your period of sick leave sufficient to cover your needs?

- Yes
- No - free text box to tell us more about why the period of sick leave was not sufficient to cover your needs.

Q16-2 Did you receive sick pay during sick leave?

- Yes, I received sick pay during all my sick leave
- Yes, I received sick pay for part of my sick leave
- No, I didn’t receive any sick pay
- Other: free text box

Q16-3 What was the reason for you not taking sick leave? (Select all that apply)

- I was not eligible for sick leave (for example, self-employed)
- I had no need for sick leave
- I would have lost pay due to sick leave
- I was on furlough due to the COVID pandemic so did not need sick leave
- My employment status changed (for example, stopped working temporarily, reduced working hours, switched to part-time employment)
- Other: free text box

Section 3: This next section is looking at how your breast cancer diagnosis may have impacted on any caring responsibilities you might have.

Q17 Do you have people who are dependent on you?

- Yes [show Q17-1]
- No
- Prefer not to say

Q17-1 Who are your dependents? (Select all that apply)

- Children
- Parents
- Partner
- Siblings
- Other: free text box
- Prefer not to say

Q18 Were you able to fulfil all your caring responsibilities **at the time you were diagnosed** and when you were undergoing treatment at hospital?

- Not applicable
- Yes
- No [show Q18-1][show Q18-2]

Q18-1 If you were not able to fulfil all your caring responsibilities **whilst undergoing treatment at hospital**, how were these responsibilities fulfilled instead? This may have included needing extra childcare or other care arrangements to allow you to attend hospital appointments or treatment. (Select all that apply)

- Some or all of my carer responsibilities were left unfilled in my absence
- Unpaid care from partner, family member, friends
- Unpaid care services from NHS, community, charity
- Paid care services

Q18-2 Please tell us more about the unfilled caring responsibilities you had and how you (and others) managed these responsibilities when you were **attending hospital appointments or treatment**.

Free text box

Q19 Are you able to fulfil all your caring responsibilities **now**?

- Not applicable
- Yes
- No [show Q19-1][show Q19-2]

Q19-1 If you are no longer able to fulfil all your caring responsibilities, how are these responsibilities fulfilled **now**? This may include needing extra childcare or other care arrangements to attend for hospital appointments, treatment or because of long-term side-effects. (Select all that apply)

- Some or all of my carer responsibilities are left unfilled in my absence
- Unpaid care from partner, family member, friends
- Unpaid care services from NHS, community, charity
- Paid care services

Q19-2 Please tell us more about the unfilled caring responsibilities you have and how you (and others) manage these responsibilities **now**.

Free text box

Section 4: We would now like to ask you about visits to and from the hospital where you have/had treatment.

Q20 How do you travel to the hospital? (Select all that apply)

- On foot
- Bicycle
- Car driven by myself
- Car driven by unpaid drivers (for example, friend, family or volunteers)
- Private car hire (for example, taxi, uber)
- Bus/tram
- Train/tube
- Hospital transport
- Other: free text box

Q21 What is the furthest you have had to travel (one-way) for treatment at the hospital?

- Less than 5 miles
- 5-20 miles
- 21-40 miles
- More than 40 miles
- Not sure

Q22 How much do you pay on average for travel per hospital visit (including parking and fuel)?

- Free
- Less than £10
- £10-30
- £31-50
- £51-100
- More than £100

Q23 Did you receive any financial support to cover your travel for treatment?

- Yes [show Q23-1] [show Q23-7]
- No [show Q23-4]

Q23-1 What best describes the financial support you received? (Select all that apply)

- A grant covered some of my transportation costs
- A grant covered all my transportation costs
- My clinical trial participation covered some of my transportation costs
- I was eligible for free public transport
- I was eligible for subsidised parking [show Q23-2]
- I was eligible for free parking [show Q23-3]

Q23-2 Did your treatment centre offer you sufficient subsidised parking?

- Yes
- No

Q23-3 Did your treatment centre offer you sufficient free parking?

- Yes
- No

Q23-4 Was it difficult to cover the costs associated with travel for treatment?

- Yes [show Q23-5]
- No

Q23-5 Were you aware of any available financial support to cover travel for treatment?

- Yes [show Q23-6]
- No

Q23-6 There are often rules to decide who gets funding for travel, for example, minimum travel distance to the hospital. Did you meet the rules for travel funding?

- Don't know, I am not aware of the rules
- Yes, I was within the rules to apply
- No, I didn't meet the rules to apply

Q23-7 Was it difficult to cover the costs associated with travel for treatment after you received financial support?

- Yes
- No

Q24 Please select all your costs related to your hospital visits: (Select all that apply)

- Travel (excluding parking)
- Parking
- Accommodation
- Miscellaneous costs (such as food and drinks)
- Loss of earnings
- Childcare
- Other care services
- Other: free text box
- None

Q25 How much do you normally spend for one hospital visit including all of the related costs (described in the question above) apart from loss of earnings?

- Less than £10
- £10-30
- £31-50
- £51-100
- More than £100

Q26 How many times did you visit the hospital every month on average for your breast cancer diagnosis and hospital-based treatment (for example, during the first 3-9 months after diagnosis)? Please enter a whole number.

Free text box

Section 5: This next section will ask about possible additional costs following a diagnosis of breast cancer.

Q27 Please select all your additional health costs related to your breast cancer in the last 6 months. (Select all that apply)

- Prescription medicine
- Dental visits
- Supportive medication/equipment to treat side-effects (for example, moisturising creams, fan for hot flushes)
- Dietary and other supplements
- Herbal remedies
- Complementary treatments
- Sport and exercise
- Private healthcare
- Care provided at home
- Supportive care (for example, massage, counselling, acupuncture, reflexology, yoga)
- Wigs, hairpieces, head coverings
- Clothing (for example, special bra, compression sleeves)
- Modification of home (for example, installing handrails) or to your car
- Other: free text box
- None

Q28 A diagnosis of cancer may change the way that people live, for example, feeling the cold more. Please select your additional costs due to breast cancer in the last month. (Select all that apply)

- Heating and fuel bills
- Food and drink
- Travel costs
- Household items
- Telephone or internet bills
- Personal care provided at home (for example, cleaning, cooking)
- Childcare
- New clothing for new body shape/needs
- New makeup, eyebrow tinting, false lashes, and other personal care
- Other: free text box
- None

Q29 Have you experienced any financial problems because of additional costs or a decrease in income?

- Yes [show Q29-1]
- No
- Prefer not to say

Q29-1 What best describes the financial problems that you have experienced? (Select all that apply)

- Loss of home
- Change of home

- Unable to pay bills
- Not able to keep up with mortgage/rent payments
- Going into debt/worsening debt
- Attending foodbanks
- Missing treatments or doctor appointments
- Change of future plans - free text box to tell us more about changes in future plans
- Other: free text box
- Prefer not to say

Q30 Do you recall your breast cancer team discussing possible financial support for you following your diagnosis of breast cancer? This may include support for travel, benefits and legal advice.

- Yes, I recall it was discussed by breast cancer team [show Q30-1]
- Not sure whether it was discussed by breast cancer team
- No, I do not recall it being discussed by breast cancer team
- No, but information was available elsewhere in the hospital

Q30-1 Was the information given at the right time for you?

- Yes
- No – free text box to tell us about when would be the right time for you.

Q31 Have you worried or found yourself worrying more about money following your breast cancer diagnosis than you did before?

- Not at all
- A little
- Quite often
- Very often
- Prefer not to say

Q32 Did you need any financial support to help cover costs associated with your breast cancer diagnosis?

- Yes [show Q32-1]
- No
- Prefer not to say

Q32-1 Did you ask for financial support?

- Yes [show Q32-2][show Q32-4][show Q32-5]
- No [show Q32-3]
- Prefer not to say

Q32-2 Where did you seek financial support from? (Select all that apply)

- Family and friends
- Fundraising (for example, Go Fund Me)
- Charity
- Government, including benefits (for example, Personal Independence Payment)
- Prefer not to say
- Other: free text box

Q32-3 Why didn't you ask for financial support?

Free text box

Q32-4 How easy was it to get financial support? (for example, finding information about the application, applying for Personal Independence Payment, completing DS1500 form)?

- Very easy
- Easy
- Difficult
- Very difficult

Q32-5 How distressing was it to apply for financial support on a scale of 0-10 (0=Not distressing at all and 10=Extremely distressing)? Please select only 1 answer per row.

Scale: 0 (Not distressing at all), 1,2,3,4,5,6,7,8,9,10 (Extremely distressing), Not applicable, Prefer not to say

Q33 Have you had problems in getting financial products such as a mortgage, bank loan, life insurance, health insurance, or travel insurance following your diagnosis?

- Not Applicable – I have not tried to get any of those
- Yes, I have had some problems getting some financial products [show Q33-1] [show Q33-2] [show Q33-3]
- No, I haven't had any problems getting financial products [go to Q33-2]
- Prefer not to say

Q33-1 Were the problems you had getting financial products related to your breast cancer diagnosis?

- Mostly related
- Somewhat related
- Mostly unrelated
- Not sure
- Prefer not to say

Q33-2 What financial product did you try to get? (Select all that apply)

- Mortgage
- Bank loan
- Life insurance
- Health insurance
- Travel insurance
- Other: free text box

Q33-3 Please can you describe the problems you had when trying to get a financial product? (for example, high price because of the breast cancer diagnosis)

Free text box

Section 6: In this next section we would like to understand your experience of clinical trials/other research.

Q34 Have you heard about clinical trials/other research opportunities?

- Yes, I have heard of such opportunities [show Q34-1]
- No, I haven't heard of such opportunities
- Not sure

Q34-1 Where did you hear about these opportunities? (Select all that apply)

- The breast cancer team
- Drug company website
- Charity or research institute website (for example, Breast Cancer Now, Cancer Research UK or The Institute of Cancer Research)
- Family and friends
- Other: free text box
- Not sure

Q35 Were you offered participation in any clinical trials/other research?

- Yes, I was offered the opportunity to participate in some clinical trials/other research [show Q35-1]
- No, I was not offered any opportunities to participate in clinical trials/other research
- Not sure

Q35-1 Did you participate in the clinical trials/other research offered to you?

- I participated in all clinical trials/other research offered [show Q35-2][Show Q35-4]
- I chose to participate in some of the clinical trials/other research offered [show Q35-2][show Q35-4][show Q35-3]
- I chose not to participate in any clinical trial/other research [show Q35-3]
- I was not eligible to participate in the clinical trials/other research

Q35-2 How was your experience of clinical trials/other research?

- Very positive
- Positive
- Negative
- Very negative

Q35-3 Which factors affected your decision to decline a clinical trial/other research: (Select all that apply)

- Extra time commitment needed
- Extra costs due to travel and hospital visits
- Relationship with the clinical teams
- Concern about possible side-effects
- Concern that treatment was new so benefit not known
- Concern about getting a placebo (inactive) treatment
- Additional information/paperwork seemed overwhelming
- Concerns from family/friends
- No specific factors
- Other: free text box

Q35-4 Which factors affected your decision to accept a clinical trial/other research (Select all that apply)

- To receive a new treatment
- To potentially help me live longer
- To potentially improve my quality of life
- To receive closer monitoring/ frequent follow-up
- To help breast cancer patients in the future
- Other: free text box

Section 7: In this next section we would like to understand how you have managed with any symptoms or difficulties related to breast cancer. Please bear in mind that different people receiving the same treatment can be affected in different ways. Links to support services will be provided at the end of the survey.

Q36 Did you experience any of the following physical or well-being issues related to your breast cancer? (Select all that apply)

- Lymphoedema
- Menopausal symptoms (for example, hot flushes, joint aches/pains)
- Impact on sexual health (for example, vaginal dryness, loss of sex drive)
- Reduction in fertility
- Anxiety
- Depression
- Memory problems
- Loss of confidence
- Concerns regarding body image
- Pain
- Nausea
- Fatigue
- Reduced mobility
- Worsening of other medical conditions
- Other: free text box
- None

Q37 Did you need help with side-effects or well-being concerns?

- Yes [show Q37-1]
- No

Q37-1 Where did you seek help for any physical or well-being issues? (Select all that apply)

- The breast cancer team
- General practitioner (GP)
- Friends or family
- Physiotherapy referred by NHS (including support for exercise)
- Private physiotherapy (including support for exercise)
- Information available from internet
- Breast cancer support groups
- Charity (for example, Breast Cancer Now and Maggie's)
- Private healthcare
- Private counsellor/psychologist
- Social services/Council (for example, for advice on support for home modifications)
- Other: free text box
- None

Q38 How would you rate the overall support you received from the NHS to manage side-effects and well-being concerns related to breast cancer on a scale of 0-10 (0=Meeting none of your needs and 10=Meeting all your needs)?

Scale: 0 (Meeting none of your needs),1,2,3,4,5,6,7,8,9,10 (Meeting all your needs), Not applicable

Q39 Did you have difficulties seeking help to manage the physical or well-being needs related to your breast cancer diagnosis?

- Not applicable
- Yes [show Q39-1]
- No

Q39-1 Please tell us about any problems you had seeking help for your physical or well-being needs.

Free text box

Q40 Did your partner or family members (including children) experience any problems with their well-being due to your breast cancer diagnosis?

- Yes [show Q40-1]
- No
- Not sure

Q40-1 Did your partner or family members (including children) need support for their well-being due to your diagnosis of breast cancer?

- Yes [show Q40-2][show Q40-3]
- No
- Not sure

Q40-2 Was enough support provided for your partner or family members (including children) for their well-being?

- Yes
- No
- Not sure

Q40-3 Please tells us more about the experience of your partner or family members (including children) in getting support for their well-being.

Free text box

Q41 Did your partner or family members (including children) experience any problems with their employment and/or finances due to your breast cancer diagnosis?

- Yes [show Q41-1]
- No
- Not sure

Q41-1 Did your partner or family members (including children) need support for their employment and/or finances due to your breast cancer diagnosis?

- Yes [show Q41-2][show Q41-3]
- No
- Not sure

Q41-2 Was there enough support for your partner or family members (including children) for any problems with their employment and/or finances due to your breast cancer diagnosis?

- Yes
- No
- Not sure

Q41-3 Please tell us more about the experience of your partner or family members (including children) in getting support for any problems with their employment and/or finances?

Free text box

Q42 Do you think there needs to be more support for partners or family members (including children) of patients with breast cancer?

- Yes[Q42-1]
- No

Q42-1 Which family members do you think need more support? (Select all that apply)

- Partners
- Children
- Parents
- Siblings
- Other: free text box

Q43 Did your breast cancer diagnosis have any effect on your relationship with your partner?

- Not applicable
- No, there were no effects
- Yes, mainly positive effects
- Yes, mainly negative effects
- Yes, some positive and some negative effects

Q44 Did your breast cancer diagnosis have any effect on your relationships with your children?

- Not applicable
- No, there were no effects
- Yes, mainly positive effects
- Yes, mainly negative effects
- Yes, some positive and some negative effects

Q45 Did your breast cancer diagnosis have any effect on your relationship with your other family members and friends?

- Not applicable
- No, there were no effects
- Yes, mainly positive effects
- Yes, mainly negative effects
- Yes, some positive and some negative effects

Q46 Please tell us how you feel about the term "cancer survivor". [show Q46-1]

- I like this term and it makes me feel positive
- I am neutral and I feel indifferent to this term
- I do not like this term and it makes me feel uncomfortable

Q46-1 Please add any other comments on how "cancer survivor" makes you feel.

Free text box

Q47 Please tell us how you feel about the term "palliative care". [show Q47-1]

- I like this term and it makes me feel positive
- I am neutral and I feel indifferent to this term
- I do not like this term and it makes me feel uncomfortable

Q47-1 Please add any other comments on how "palliative care" makes you feel.

Free text box

Supportive cancer care is prevention and management of the effects of cancer and its treatment. This includes management of physical symptoms and well-being during the whole cancer journey from diagnosis through treatment to after-care. Supportive care aims to improve rehabilitation, secondary cancer prevention, survivorship, and end-of-life care.

Q48 Have you ever heard of "supportive care"?

- Yes
- No

Q49 How does "supportive care" make you feel?

- I like this term and it makes me feel positive
- I am neutral and I feel indifferent to this term
- I do not like this term and it makes me feel uncomfortable

Q50 Please tell us about your opinion on the following statement: "Supportive care includes all the support I need during and after my breast cancer treatment."

- Strongly agree
- Agree
- Neutral
- Disagree
- Strongly disagree

Q50-1 Please add any other comments on how "supportive care" makes you feel.

Free text box

Q51 How do you think you are doing at the moment? Please select only 1 answer per row.

- From a physical point of view
- From a well-being point of view
- From a financial point of view

Scale: Very poorly, Poorly, Well, Very Well, Prefer not to say

Q52 How much do you think your breast cancer has impacted your everyday activities on a scale of 0-10 (0=Very little and 10=Very much)? Please select only 1 answer per row.

- Caring
- Employment
- Domestic activities
- Volunteering
- Hobbies
- Exercise
- Holidays
- Overall

Scale: 0 (Very little),1,2,3,4,5,6,7,8,9,10 (Very much), Not applicable, Prefer not to say

Q53 How optimistic do you feel about the future on a scale of 0-10 (0=Not very optimistic and 10=Very optimistic)? Please select only 1 answer per row.

Scale:0 (Not very optimistic),1,2,3,4,5,6,7,8,9,10 (Very optimistic), Prefer not to say

Q54 Please feel free to tell us about anything else that you think we have missed.

Free text box

Section 8: We are now near the end and have a few final questions about you to help us analyse the results. This information will help us get a better idea of how individuals from different backgrounds across the UK have managed with the issues we have discussed in this survey.

Q55 What was your age **at the time of your diagnosis**? (Required question)

- Below 30 years
- 30-40 years
- 41-50 years
- 51-60 years
- 61-70 years
- 71-80 years
- 81 years and above
- Prefer not to say

Q56 What is the first part of your postcode (for example, SE3, N8, EC3W)? If your postcode has changed since the breast cancer diagnosis (and treatment), please provide us with the first part of your postcode when you were undergoing breast cancer treatment.

____

Q57 What is your sex? (Required question)

- Female
- Male
- Other: fee text box
- Prefer not to say

Q58 Is the gender you identify with the same as your sex registered at birth? (Required question)

- Yes
- No
- Prefer not to say

Q59 Which of the following best describes your sexual orientation? (Required question)

- Straight/Heterosexual
- Gay or Lesbian
- Bisexual
- Other sexual orientation: free text box
- Prefer not to say

Q60 What was your marital status **at the time you were diagnosed**? (Required question) [show Q60-1]

- Single
- Married/Cohabiting
- Divorced/Separated
- Widowed
- Prefer not to say

Q60-1 Have there been any changes to your marital status since you were diagnosed? (Required question)

- Yes [show Q60-2]
- No
- Prefer not to say

Q60-2 What is your marital status **now**?<br>• Single<br>• Married/Cohabiting<br>• Divorced/Separated<br>• Widowed<br>• Prefer not to say
Q61 Were you living alone **at the time you were diagnosed**? (Required question) [show Q61-1]<br>• Yes<br>• No<br>• Prefer not to say<br>Q61-1 Have there been any changes to your living arrangements since you were diagnosed? (Required question)<br>• Yes<br>• No<br>• Prefer not to say<br>Q61-2 Are you living alone **now**?<br>• Yes<br>• No<br>• Prefer not to say
Q62 What is your ethnic group? Please choose one option that best describes your ethnic group or background. (Required question)<br>• White [show Q62-1]<br>• Mixed or Multiple ethnic groups [show Q62-2]<br>• Asian or Asian British [show Q62-3]<br>• Black, Black British, Caribbean or African [show Q62-4]<br>• Other ethnic group [show Q62-5]<br>• Prefer not to say<br>Q62-1 Please choose one option that best describes your ethnic group or background.<br>• English, Welsh, Scottish, Northern Irish or British<br>• Irish<br>• Gypsy or Irish Traveller<br>• Roma<br>• Other: free text box<br>• Prefer not to say<br><br>Q62-2 Please choose one option that best describes your ethnic group or background.<br>• White and Black Caribbean<br>• White and Black African<br>• White and Asian<br>• Other: free text box<br>• Prefer not to say<br><br>Q62-3 Please choose one option that best describes your ethnic group or background.<br>• Indian<br>• Pakistani<br>• Bangladeshi<br>• Chinese<br>• Other: free text box<br>• Prefer not to say

Q62-4 Please choose one option that best describes your ethnic group or background.<br>• Caribbean<br>• African background: free text box<br>• Other: free text box<br>• Prefer not to say<br><br>Q59-5 Please choose one option that best describes your ethnic group or background.<br>• Arab<br>• Any other ethnic group: free text box<br>• Prefer not to say
Q63 What is your religion? (Required question)<br>• No religion<br>• Christian (including Church of England, Catholic, Protestant and all other Christian denominations)<br>• Buddhist<br>• Hindu<br>• Jewish<br>• Muslim<br>• Sikh<br>• Other: free text box<br>• Prefer not to say
Q64 Please select your highest educational level (Required question)<br>• Postgraduate degree/degree/professional qualification<br>• A level/HND or equivalent<br>• School certificate/GCSE/O-level/NVQ or equivalent<br>• Other: free text box<br>• None<br>• Prefer not to say

Thank you for taking the time to complete this survey.

You can find more information about Breast Cancer Now services support at the following webpage: https://breastcancernow.org/information-support/support-you
