# Supplementary material for: Hidden costs and unmet supportive care needs among individuals with experience of breast cancer and their carers in the United Kingdom
Source: BJC Rep. 2025 Aug 19;3:55. doi: 10.1038/s44276-025-00172-z (PMC12361448; doi:10.1038/s44276-025-00172-z)
Supplement: Supplementary file 3 — Supplementary Material 2 CASCARA carer survey [file 44276_2025_172_MOESM3_ESM.pdf]

CASCARA (Costs And Supportive Care in bReast cAncer) survey (for relatives/friends who have cared for someone with breast cancer)

Introduction

The CASCARA research team are investigating the hidden financial costs and supportive care needs associated with breast cancer. This work is part of the Lancet Breast Cancer Commission which is looking at worldwide problems in breast cancer and how we can address them. We hope that by raising awareness of these hidden costs and supportive care needs, we can drive change towards better support for people with breast cancer and their families and friends who care for them.

In this survey we would like to hear from relatives or friends who care or have cared for someone with breast cancer who received their treatment in the UK. The survey should take about 5-10 minutes to complete. Your answers will be completely anonymous. Thank you so much for taking the time to complete this survey – your views are important and may help others in the future.

You can review our GDPR privacy statement on our website: https://www.icr.ac.uk/legal/privacy

If you have any questions about this research, please feel free to email any questions to the CASCARA team: **cascara-icrctsu@icr.ac.uk**

If you would be happy to take part, please click **Next** to continue.

Throughout this survey, we will refer to the person with breast cancer that you care for or have cared for as your relative/friend.

If your relative/friend has had more than one diagnosis of breast cancer or the cancer has returned, please provide information related to the most recent experience.

We will refer to you as a carer. If you have more than one dependant to care for, please provide information related to becoming a carer for your relative/friend with breast cancer.

Section 1: We would like to ask about your relative/friend's diagnosis of breast cancer.

Q1 What is your relationship to your relative/friend? (Required question)

- Partner
- Parent
- Child
- Sibling
- Friend
- Other: free text box

Q2 When your relative/friend was most recently treated for breast cancer, was it for: (Required question)

- Primary (early) breast cancer (where cancer is only in the breast and/or lymph nodes)
- Secondary (metastatic) breast cancer (where breast cancer has spread to other parts of the body)
- Not sure

Q3 When was your relative/friend's diagnosis of breast cancer? (Required question)

- Within the last 1 year
- 1 year ago
- 2 years ago
- 3 years ago
- 4 years ago
- 5 years ago
- More than 5 years ago
- Not sure

Q4 Where are they currently in the treatment pathway? (Required question)

- Awaiting first treatment
- Undergoing treatment at hospital (for example, surgery, radiotherapy, chemotherapy, targeted therapy)
- Completed treatment at hospital (for example, surgery, radiotherapy, chemotherapy, targeted therapy) and receiving ongoing treatment at home (for example, hormone therapy, ovarian suppression, bisphosphonates)
- Completed all treatment
- Not sure
- Other: free text box

Section 2: We would like to find out more about your employment and how caring for someone with breast cancer may have affected your employment.

Q5 What was your work status **when you became a carer**?

- Full time employment [show Q5-2][show Q5-3][show Q5-4]
- Part time employment [show Q5-2][show Q5-3][show Q5-4]
- Not in paid employment [show Q5-1]
- Retired
- Prefer not to say

Q5-1 Were you looking for employment **when you became a carer**?

- Yes
- No
- Prefer not to say

Q5-2 On average, how many hours did you work per week **when you became a carer**? Please enter a whole number.

Free text box

Q5-3 How would you describe your work schedule **when you became a carer**?

- Fixed working hours (not able to adjust start and finish times)
- Flexible working hours (able to adjust start and finish times)
- Prefer not to say

Q5-4 Were you self-employed **when you became a carer**?

- Yes
- No
- Prefer not to say

Q6 Were you or your partner receiving any benefits including universal credit **when you became a carer**?

- Yes [show Q6-1]
- No
- Prefer not to say

Q6-1 If you or your partner were receiving any benefits **when you became a carer**, please can you tell us what benefits you were receiving?

Free text box

Q7 What was your estimated annual personal income pre-tax (including take home pay, benefits or any other regular income) **when you became a carer**?

- Less than £12,570
- £12,570-25,000
- £25,001-50,000
- £50,001-75,000
- £75,001-100,000
- More than £100,000
- Prefer not to say

Q8 What is your work status **now**?

- Full time employment [show Q8-2][show Q8-3][show Q8-4]
- Part time employment [show Q8-2][show Q8-3][show Q8-4]
- Not in paid employment [show Q8-1]
- Retired
- Prefer not to say

Q8-1 Are you looking for employment **now**?

- Yes
- No
- Prefer not to say

Q8-2 On average, how many hours do you work per week **now**? Please enter a whole number.

Free text box

Q8-3 How would you describe your work schedule **now**?

- Fixed working hours (not able to adjust start and finish times)
- Flexible working hours (able to adjust start and finish times)
- Prefer not to say

Q8-4 Are you self-employed **now**?

- Yes
- No
- Prefer not to say

Q9 Are you or your partner receiving any benefits including universal credit **now**?

- Yes [show Q9-1]
- No
- Prefer not to say

Q9-1 If you or your partner are receiving any benefits **now**, please can you tell us what benefits you receive?

Free text box

Q10 What is your estimated annual personal income pre-tax (including take home pay, benefits or any other regular income) **now**?

- Less than £12,570
- £12,570-25,000
- £25,001-50,000
- £50,001-75,000
- £75,001-100,000
- More than £100,000
- Prefer not to say

Q11 If there have been changes in your employment since you became a carer, how much was this influenced by your caring role?

- Not applicable
- Not at all
- Slightly [Show Q11-1][Show Q11-2]
- Somewhat [Show Q11-1][Show Q11-2]
- Very much [Show Q11-1][Show Q11-2]

Q11-1 Overall, how do you feel about the changes in your employment since you became a carer?

- Mostly happy
- Somewhat happy
- Somewhat unhappy
- Mostly unhappy

Q11-2 Please can you tell us how your caring role has influenced your employment?

Free text box

Q12 To what extent do you agree with the following statement: "I have been treated fairly at work since my caring role started."

- Not applicable
- Strongly agree
- Agree
- Neutral
- Disagree
- Strongly disagree
- Prefer not to say

Q13 To what extent do you agree with the following statement: "I have been discriminated against at work due to my caring role."

- Not applicable
- Strongly agree
- Agree
- Neutral
- Disagree
- Strongly disagree

- Prefer not to say

Q14 Did you take compassionate/carer’s leave?

- Not applicable (for example, not in paid employment)
- Yes [show Q14-1][show Q14-2]
- No [show Q14-3]

Q14-1 Was your period of compassionate/carer’s leave sufficient to cover your needs?

- Yes
- No - free text box to tell us more about why the period of compassionate/carer’s leave was not sufficient to cover your needs.

Q14-2 Did you receive any pay during compassionate/carer’s leave?

- Yes, I received pay during all my compassionate/carer’s leave
- Yes, received pay for part of my compassionate/carer’s leave
- No, I didn’t receive any pay
- Other: free text box

Q14-3 What was the reason for you not taking compassionate/carer’s leave? (Select all that apply)

- I was not eligible for compassionate/carer’s leave (for example, self-employed)
- I had no need for compassionate/carer’s leave
- I would have lost pay due to compassionate/carer’s leave
- I was on furlough due to the COVID pandemic and so did not need compassionate/carer’s leave
- Changed employment status (for example, stopped working temporarily, reduced working hours, switched to part-time employment)
- Other: free text box

Section 3: This next section is looking at how your caring role for your relative/friend with breast cancer may have affected other caring roles you might have.

Q15 Do you have people who were dependent on you before you became a carer for your relative/friend?

- Yes [show Q15-1]
- No
- Prefer not to say

Q15-1 Who are your dependents? (Select all that apply)

- Children
- Parents
- Partner
- Siblings
- Other: free text box
- Prefer not to say

Q16 Were you able to fulfil all your other caring responsibilities when your relative/friend had hospital appointments?

- Not applicable
- Yes
- No [show Q16-1] [show Q16-2]

Q16-1 If you were not able to fulfil all your other caring responsibilities when your relative/friend was undergoing treatment at hospital, how were these responsibilities fulfilled instead? (Select all that apply)

- Some or all of my carer responsibilities were left unfilled in my absence
- Unpaid care from partner, family member, friends
- Unpaid care services from NHS, community, charity
- Paid care services

Q16-2 Please tell us more about the unfilled caring responsibilities you had and how you (and others) managed these caring responsibilities when your relative/friend was attending for hospital appointments or treatment.

Free text box

Section 4: In this section we would like to find out more about your supportive care needs.

Q17 Did you experience any well-being issues since becoming a carer?

- Yes [show Q17-1]
- No

Q17-1 Did you need support with your well-being issues since becoming a carer?

- Yes [show Q17-2] [show Q17-3]
- No

Q17-2 Was there enough support for your well-being?

- Yes
- No

Q17-3 Please tell us more about your experience in getting support for your well-being.

Free text box

Q18 Have you had any problems with finances as a result of your caring role?

- Yes [show Q18-1]
- No

Q18-1 Did you need support with your finances due to your caring role?

- Yes [show Q18-2] [Q18-3]
- No

Q18-2 Was there enough support provided to help you with your finances due to your caring role?

- Yes
- No

Q18-3 Please tell us about your experience in getting support to help with your finances due to your caring role.

Free text box

Q19 Do you think there needs to be more support for carers for people with breast cancer?

- Yes [show Q19-1]
- No

Q19-1 Please tell us more about the support you think is needed for carers for people with breast cancer.

Free text box

Q20 Has the relationship with your relative/friend been affected since you became their carer?

- Not applicable
- No, there were no effects
- Yes, mainly positive effects
- Yes, mainly negative effects
- Yes, some positive and some negative effects

Q21 How much do you think your caring role has affected your everyday activities on a scale of 0-10 (0=Very little and 10=Very much)? Please select only 1 answer per row.

- Other caring responsibilities
- Employment
- Domestic activities
- Volunteering
- Hobbies
- Exercise
- Holidays
- Overall

Scale: 0 (Very little),1,2,3,4,5,6,7,8,9,10 (Very much), Not applicable, Prefer not to say

Q22 How optimistic do you feel about the future as a carer for someone with breast cancer on a scale of 0-10 (0=Not very optimistic and 10=Very optimistic)? Please select only 1 answer per row.

Scale: 0 (Not very optimistic),1,2,3,4,5,6,7,8,9,10 (Very optimistic), prefer not to say

Q23 Please feel free to tell us about anything else that you think we have missed about your experience of being a carer for someone with breast cancer.

Free text box

Section 5: We are now near the end and have a few final questions about you to help us analyse the results. This information will help us get a better idea of how individuals from different backgrounds across the UK have managed with the issues we have discussed in this survey.

Q24 What was your age at the time when you became a carer for your relative/friend? (Required question)<br>• Below 30 years<br>• 30-40 years<br>• 41-50 years<br>• 51-60 years<br>• 61-70 years<br>• 71-80 years<br>• 81 years and above<br>• Prefer not to say
Q25 What is the first part of your postcode (for example, SE3, N8, EC3W)? If your postcode has changed since the breast cancer diagnosis (and treatment), please provide us with the first part of your postcode when you became a carer for your relative/friend.<br>Free text box
Q26 What is your sex? (Required question)<br>• Female<br>• Male<br>• Other: free text box<br>• Prefer not to say
Q27 Is the gender you identify with the same as your sex registered at birth? (Required question)<br>• Yes<br>• No<br>• Prefer not to say
Q28 Which of the following best describes your sexual orientation? (Required question)<br>• Straight/Heterosexual<br>• Gay or Lesbian<br>• Bisexual<br>• Other sexual orientation: free text box<br>• Prefer not to say
Q29 What was your marital status **when you became a carer for your relative/friend**? (Required question) [show Q29-1]<br>• Single<br>• Married/Cohabiting<br>• Divorced/Separated<br>• Widowed<br>• Prefer not to say<br>Q29-1 Have there been any changes to your marital status since you became a carer for your relative/friend? (Required question)<br>• Yes [show Q29-2]<br>• No<br>• Prefer not to say<br>Q29-2 What is your marital status **now**?

- Single
- Married/Cohabiting
- Divorced/Separated
- Widowed
- Prefer not to say

Q30 Were you living alone **when you became a carer for your relative/friend**? (Required question) [show Q30-1]

- Yes
- No
- Prefer not to say

Q30-1 Have there been any changes to your living arrangements since you became a carer for your relative/friend? (Required question)

- Yes [show Q30-2]
- No
- Prefer not to say

Q30-2 Are you living alone **now**?

- Yes
- No
- Prefer not to say

Q31 What is your ethnic group? Please choose one option that best describes your ethnic group or background. (Required question)

- White [show Q31-1]
- Mixed or Multiple ethnic groups [show Q31-2]
- Asian or Asian British [show Q31-3]
- Black, Black British, Caribbean or African [show Q31-4]
- Other ethnic group [show Q31-5]
- Prefer not to say

Q31-1 Please choose one option that best describes your ethnic group or background.

- English, Welsh, Scottish, Northern Irish or British
- Irish
- Gypsy or Irish Traveller
- Roma
- Other: free text box
- Prefer not to say

Q31-2 Please choose one option that best describes your ethnic group or background.

- White and Black Caribbean
- White and Black African
- White and Asian
- Other: free text box
- Prefer not to say

Q31-3 Please choose one option that best describes your ethnic group or background.

- Indian
- Pakistani
- Bangladeshi
- Chinese

- Other: free text box
- Prefer not to say

Q31-4 Please choose one option that best describes your ethnic group or background.

- Caribbean
- African background: free text box
- Other: free text box
- Prefer not to say

Q31-5 Please choose one option that best describes your ethnic group or background.

- Arab
- Other: free text box
- Prefer not to say

Q32 What is your religion? (Required question)

- No religion
- Christian (including Church of England, Catholic, Protestant and all other Christian denominations)
- Buddhist
- Hindu
- Jewish
- Muslim
- Sikh
- Other: free text box
- Prefer not to say

Q33 Please select your highest educational level (Required question)

- Postgraduate/Degree/Professional qualification
- A level/HND or equivalent
- School certificate/GCSE/O-level/NVQ or equivalent
- None
- Other: free text box
- Prefer not to say

Thank you for taking the time to complete this survey.

You can find more information about support for friends and family caring for someone with breast cancer at Section 3 on the following webpage: https://breastcancernow.org/information-support/facing-breast-cancer/how-support-someone-breast-cancer#you
